# Hydroxycitrate delays early mortality in mice and promotes muscle regeneration while inducing a rich hepatic energetic status

**DOI:** 10.1111/acel.14205

**Published:** 2024-05-17

**Authors:** Isabel Espadas, María Ángeles Cáliz‐Molina, Raúl López‐Fernández‐Sobrino, Concepción Panadero‐Morón, Alejandro Sola‐García, Mario Soriano‐Navarro, Enrique Martínez‐Force, Mónica Venegas‐Calerón, Joaquin J. Salas, Franz Martín, Benoit R. Gauthier, Clara Alfaro‐Cervelló, David Martí‐Aguado, Vivian Capilla‐González, Alejandro Martín‐Montalvo

**Affiliations:** ^1^ Andalusian Molecular Biology and Regenerative Medicine Centre‐CABIMER Universidad de Sevilla‐CSIC‐Universidad Pablo de Olavide Seville Spain; ^2^ Electron Microscopy Core Facility, Centro de Investigación Príncipe Felipe (CIPF) Valencia Spain; ^3^ Instituto de la Grasa (CSIC) Universidad Pablo de Olavide Sevilla Spain; ^4^ Biomedical Research Network on Diabetes and Related Metabolic Diseases‐CIBERDEM Instituto de Salud Carlos III Madrid Spain; ^5^ Pathology Department, INCLIVA Health Research Institute, Clinic University Hospital University of Valencia Valencia Spain; ^6^ Digestive Disease Department, Clinic University Hospital INCLIVA Health Research Institute Valencia Spain; ^7^ Division of Gastroenterology, Hepatology and Nutrition Center for Liver Diseases

**Keywords:** ACLY, health span, hydroxycitrate, lifespan, liver, muscle strength, tissue regeneration

## Abstract

ATP citrate lyase (ACLY) inhibitors have the potential of modulating central processes in protein, carbohydrate, and lipid metabolism, which can have relevant physiological consequences in aging and age‐related diseases. Here, we show that hepatic phospho‐active ACLY correlates with overweight and Model for End‐stage Liver Disease score in humans. Wild‐type mice treated chronically with the ACLY inhibitor potassium hydroxycitrate exhibited delayed early mortality. In AML12 hepatocyte cultures, the ACLY inhibitors potassium hydroxycitrate, SB‐204990, and bempedoic acid fostered lipid accumulation, which was also observed in the liver of healthy‐fed mice treated with potassium hydroxycitrate. Analysis of soleus tissue indicated that potassium hydroxycitrate produced the modulation of wound healing processes. In vivo, potassium hydroxycitrate modulated locomotor function toward increased wire hang performance and reduced rotarod performance in healthy‐fed mice, and improved locomotion in mice exposed to cardiotoxin‐induced muscle atrophy. Our findings implicate ACLY and ACLY inhibitors in different aspects of aging and muscle regeneration.

## Graphical abstract

ATP citrate lyase (ACLY) inhibitors have been proposed as calorie restriction mimetics. We performed a longevity assay in mice using the ACLY inhibitor hydroxycitrate. We observed that hydroxycitrate delays early mortality, having pleiotropic effects in muscle and liver physiology. Hydroxycitrate produced an energy‐rich status in the liver, while modulated pathways involved in muscle regeneration and potentiated locomotor function in mice challenged with the myonecrotic agent carditoxin.

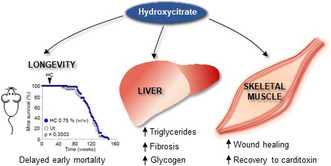

**Abbreviations**
Ac‐CoA: acetyl coenzyme A
ACLY: ATP‐citrate lyase
Acss2: cytosolic Acyl‐coenzyme A synthetase short‐chain family member 2
ALT: alanine transaminase
AST: aspartate transaminase
BAT: brown adipose tissue
Bemp: Bempedoic acid
BMI: body mass index
CSA: cross‐sectional area
CTX: cardiotoxin
DPI: days post injury
ECAR: extracellular acidification rate
FAMES: fatty acid methyl esters
GSEA: gene Set Enrichment Analysis
HC: potassium hydroxycitrate
HCA: hydroxycitric acid
HFD: high fat diet
HOMA‐IR: homeostatic assessment of insulin resistance
INR: international normalized ratio
IPA: ingenuity pathway analysis
IPPTT: intraperitoneal pyruvate tolerance test
ITT: insulin tolerance test
MELD: model for End‐stage Liver Disease
OCR: oxygen consumption rate
OGTT: oral glucose tolerance test
PAS: periodic acid Schiff
PCA: principal component analysis
RXR: retinoid X receptors
SB: SB‐204990
SEM: standard error of the mean
STD: standard diet
T4: thyroxine
TNF: tumor necrosis factor
WAT: white adipose tissue

## 1 INTRODUCTION

Unhealthy aging is a major public health challenge. During the last decades, life expectancy has increased considerably, but in many cases this increase in longevity is not accompanied by an optimal quality of life during old age and ~50% of people older than 80 years of age suffer dependency (Kalyani et al., 2014). Given the high incidence of pathologies associated with aging (e.g., sarcopenia, diabetes, neurodegenerative diseases and cancer, among others), there is an urgent need to develop therapies to prevent and treat these conditions and promote active healthy aging.

ATP citrate lyase (ACLY) catalyzes the production of cytosolic acetyl coenzyme A (Ac‐CoA) and oxaloacetate from coenzyme A and citrate in the presence of ATP. ACLY is a particularly interesting target in aging and age‐related diseases due to its ability to modulate critical cellular tasks, such as cholesterol synthesis, coenzyme Q synthesis, de novo lipogenesis, hepatic gluconeogenesis, and protein acetylation, among others (Granchi, 2018). The lack of ACLY activity and ACLY inhibitors exert calorie restriction‐like effects, such as the potentiation of immunosurveillance and autophagic flux (Mariño et al., 2014; Pietrocola et al., 2016), implicating ACLY activity and ACLY inhibitors in complex processes that have to be properly coordinated to sustain optimal health span and lifespan. Intriguingly, in certain cases, naturally occurring ACLY inhibitors, such as Garcinia extracts, rich in hydroxycitric acid (HCA), have been shown to produce hepatotoxic effects even in humans (Zovi et al., 2022). However, these extracts are a mixture of chemicals, which impedes the assessment of the toxicity of HCA, as other components in the formulation may cause the adverse effects. These evidences raise the need of further delineating the therapeutic potential of ACLY inhibition.

Despite the large body of research using ACLY inhibitors, the chronic effects of these compounds in relevant aspects of health span and lifespan have not been investigated in mammals. In this report, we set out to investigate the chronic effects of ACLY inhibition in neurocognitive health, metabolic health, physical health, lifespan, and we evaluated tissue specific effects in liver and skeletal muscle. We exposed chronically wild‐type mice to potassium hydroxycitrate (HC) using a healthy diet, and we evaluated the effects of three extensively used ACLY inhibitors, HC, SB‐204990 (SB), and bempedoic acid (Bemp) in cell cultures. Overall, our results indicate that HC delays early mortality and modulates liver and muscle physiology in healthy mice, exerting pro‐regenerative benefits in an experimental model of skeletal muscle atrophy.

## 2 RESULTS

### 2.1 Hepatic phospho‐ACLY levels correlate with obesity and liver disease

**FIGURE 1:**
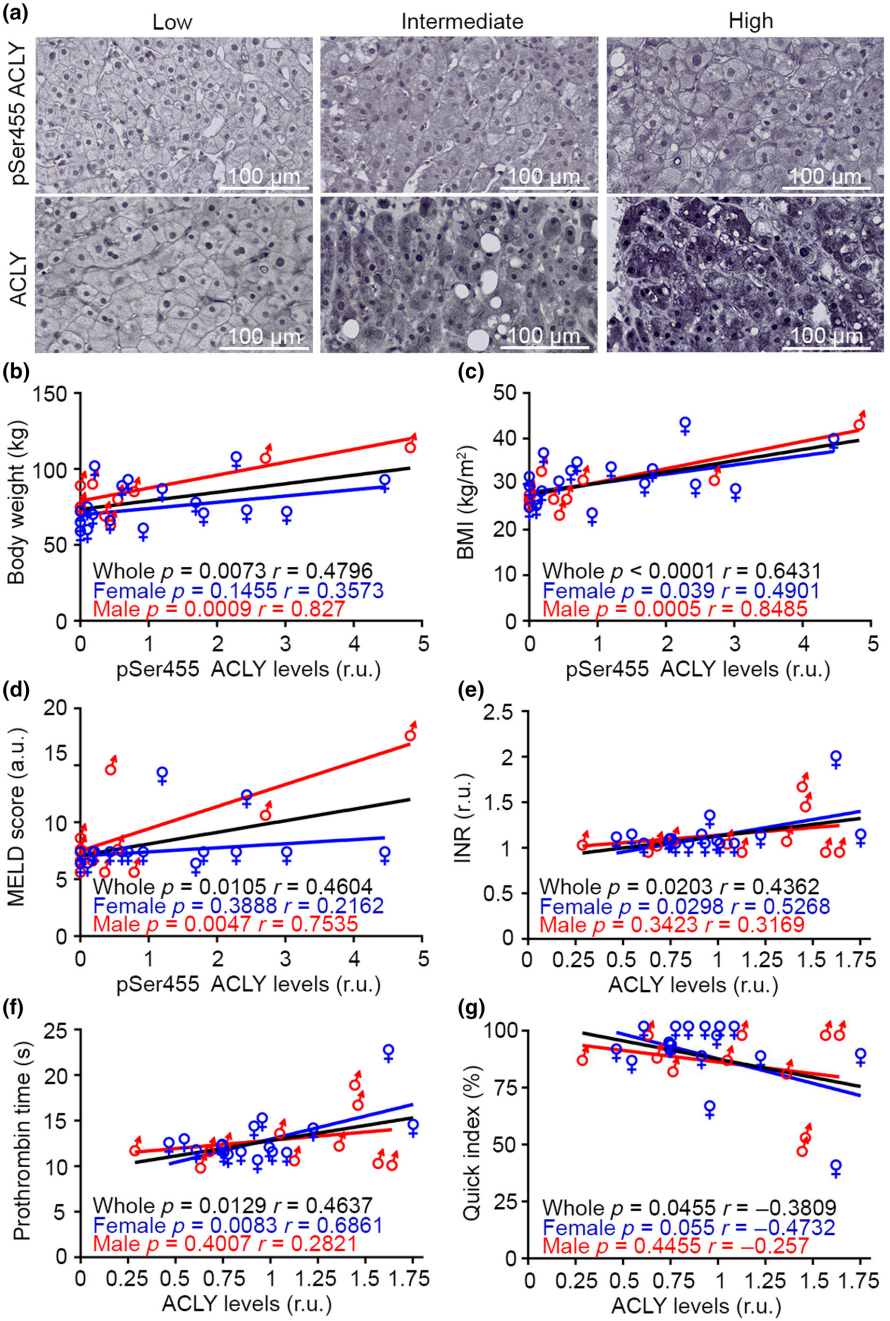
Hepatic phospho‐active ACLY expression is positively correlated with overweight and MELD score in humans. (a) Representative staining of pSer455 ACLY and total ACLY in patients undergoing liver biopsies. pSer455 ACLY low: body weight 56 kg, BMI 23.9, MELD 7. pSer455 ACLY intermediate: body weight 84 kg, BMI 32.8, MELD 14. pSer455 ACLY high: body weight 117 kg, BMI 44, MELD 18. ACLY low: INR 1.08, prothrombin time 12.2 s, Quick index 89. ACLY intermediate: INR 1.31, prothrombin time 14.8 s, Quick index 65. ACLY high: INR 1.96, prothrombin time 22.3 s, Quick index 39; *n* = 12 males and 18 females. (b) Linear regression of hepatic pSer455 ACLY and body weight. *n* = 12 males and 18 females. Pearson correlation. (c) Linear regression of hepatic pSer455 ACLY and BMI; *n* = 12 males and 18 females; Pearson correlation. (d) Linear regression of hepatic pSer455 ACLY and MELD; *n* = 12 males and 18 females; Pearson correlation. (e) Linear regression of hepatic ACLY and international normalized ratio; *n* = 11 males and 17 females; Pearson correlation. (f) Linear regression of hepatic ACLY and prothrombin time; *n* = 11 males and 17 females; Pearson correlation. (g) Linear regression of hepatic ACLY and Quick index; *n* = 11 males and 17 females; Pearson correlation. a.u., arbitrary units; INR, international normalized ratio; MELD, Model for End‐stage Liver Disease; r.u., relative units. Data shown are individual biological values.

We have previously described that hepatic ACLY expression and activity is increased in aged healthy‐fed wild‐type mice (Sola‐García et al., 2023). However, the relevance of ACLY expression levels in somatic tissues (i.e., non‐tumor) in humans has not been investigated in depth. In order to evaluate whether alterations in ACLY levels are associated with organismal health, we evaluated by immunohistochemical analyses a cohort of 30 individuals that underwent liver biopsies for their clinical diagnosis. We performed an unbiased analysis in which each biopsy was evaluated in a blinded manner by an experienced pathologist and quantified for intensity of staining for total ACLY and phospho‐active pSer455 ACLY. A striking positive correlation was observed between phospho pSer455 ACLY and body weight, body mass index (BMI), and Model for End‐stage Liver Disease (MELD) score in the whole human population studied (Figure 1a–d). The correlation of BMI with phospho‐ACLY was significant in both sexes, whereas the effects observed in the whole population were mostly supported by the male gender in the case of body weight and MELD score. We also found sex‐specific inverse correlations of phospho‐ACLY levels with free thyroxine (T4) and digital pathology (% collagen) (Marti‐Aguado et al., 2021) in male subjects, and specific negative correlations of phospho‐ACLY levels with alanine transaminase (ALT) levels in female subjects (Figure S1A–C). Total ACLY levels were correlated with international normalized ratio (INR), prothrombin time and quick index, reflective of negative alterations in the hepatic control of the coagulation system in individuals expressing high levels of ACLY in the whole population of the study (Figure 1e–g). In these correlations, the effects on the whole population were mostly supported by the female gender. Sex‐specific correlations were found for circulating levels of ferritin and the Deugnier iron score in the case of male subjects and the percentage of lymphocytes in female individuals (Figure S1D–F). In the case of females, significant higher levels of hepatic ACLY were also found in hypothyroid patients (Figure S1G). Remarkably, we determined a weak trend in the positive correlation of hepatic pSer455 ACLY and ACLY levels that did not reach statistical significance, suggesting that the translational control of ACLY expression is separated from signaling events that promote full ACLY activation (Figure S1H). Overall, these results indicate that increased hepatic phospho‐active and total ACLY levels correlate with obesity and hepatic alterations, being detrimental for human health.

### 2.2 ACLY inhibitors increase lipid content and reduce mitochondrial metabolism

**FIGURE 2:**
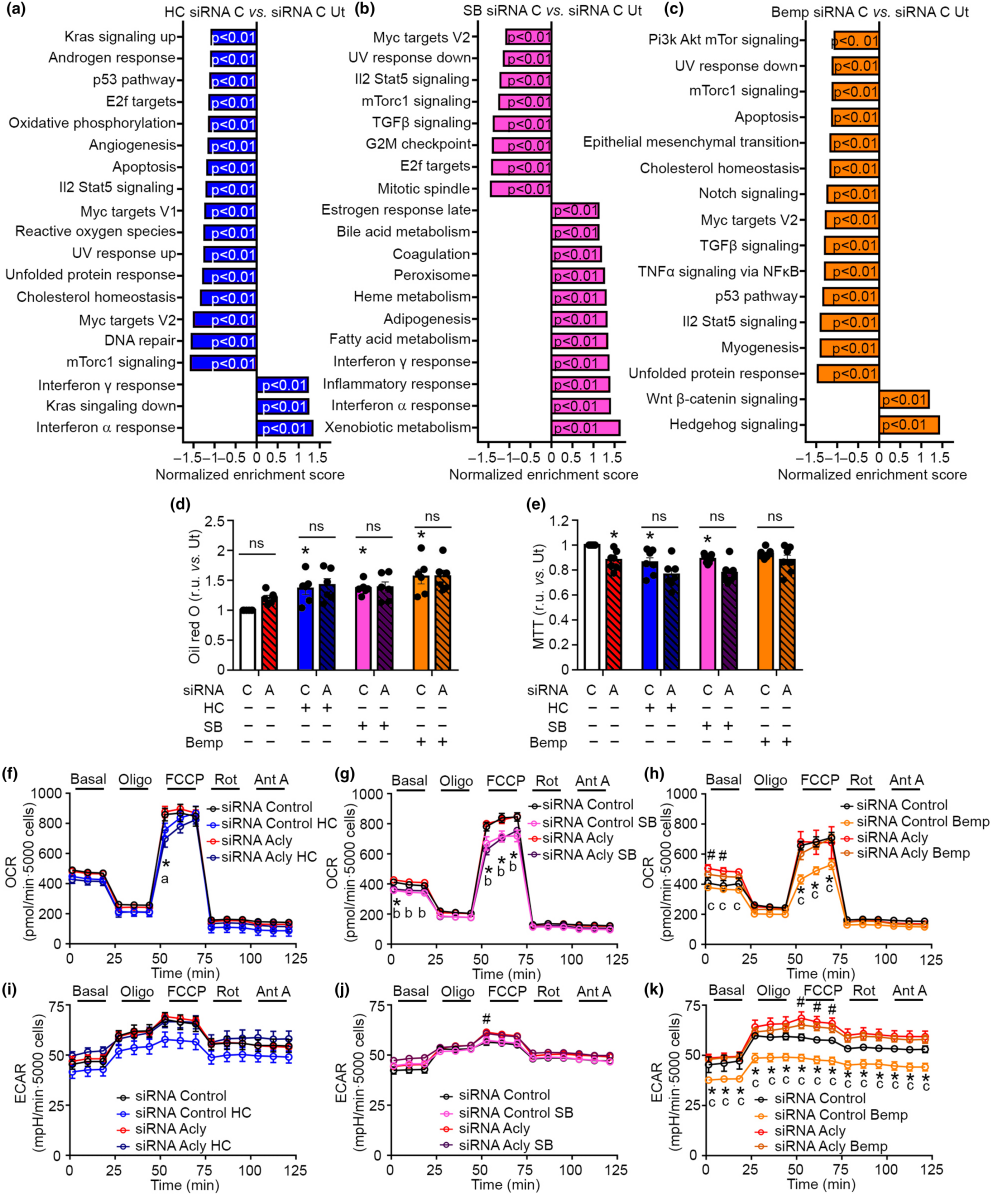
ACLY inhibitors increase lipid content and reduce metabolic activity. AML12 cells were siRNA interfered for *ACLY* expression and were treated with HC 1 mM, SB 10 μM, or Bemp 30 μM for 16 h. (a) Enriched gene sets by GSEA analysis in siRNA control HC vs. siRNA control. *n* = 3. (b) Enriched gene sets by GSEA analysis in siRNA control SB vs. siRNA control. *n* = 3. (c) Enriched gene sets by GSEA analysis in siRNA control Bemp vs. siRNA control. *n* = 3. (d) Lipid determination by Oil red O in AML12 cells. *n* = 6. Kruskal–Wallis Dunn's post hoc test. (e) Metabolic activity by MTT test. *n* = 7. Kruskal–Wallis plus Dunn's post hoc test. (f) OCR of cells treated with HC. *n* = 5 siRNA Control Ut, *n* = 5 siRNA Control HC, *n* = 3 siRNA ACLY Ut, *n* = 3 siRNA ACLY HC. Two‐way ANOVA plus Tukey post hoc test. (g) OCR of cells treated with SB. *n* = 5. Two‐way ANOVA plus Tukey post hoc test. (h) OCR of cells treated with Bemp. *n* = 5 siRNA Control Ut, *n* = 5 siRNA Control Bemp, *n* = 5 siRNA ACLY Ut, *n* = 5 siRNA ACLY Bemp. Two‐way ANOVA plus Tukey post hoc test. (i) ECAR of cells treated with HC. *n* = 5 siRNA Control Ut, *n* = 5 siRNA Control HC, *n* = 3 siRNA ACLY Ut, *n* = 3 siRNA ACLY HC. Two‐way ANOVA plus Tukey post hoc test. (j) ECAR of cells treated with SB. *n* = 5. Two‐way ANOVA plus Tukey post hoc test. (k) ECAR of cells treated with Bemp. *n* = 5 siRNA Control Ut, *n* = 5 siRNA Control Bemp, *n* = 4 siRNA ACLY Ut, *n* = 5 siRNA ACLY Bemp. Two‐way ANOVA plus Tukey post hoc test. A, siRNA ACLY; Ant A, antimycin A; Bemp, bempedoic acid; C, siRNA control; FCCP, carbonyl cyanide 4‐(trifluoromethoxy) phenylhydrazone; HC, Hydroxycitrate; ns, not significant; Oligo, oligomycin; Rot, rotenone; SB, SB‐204990; Ut, untreated. Data shown are the means ± SEM. *p < 0.05 HC vs. Ut or HC/SB/Bemp treatment in siRNA control cells vs. siRNA control Ut cells. ^#^ *p* < 0.05 siRNA ACLY Ut vs. siRNA Control Ut. ^a^ *p* < 0.05 siRNA ACLY HC vs. siRNA ACLY Ut. ^b^ *p* < 0.05 siRNA ACLY SB vs. siRNA ACLY Ut. ^c^ *p* < 0.05 siRNA ACLY Bemp vs. siRNA ACLY Bemp.

Given the implications of increased phospho‐ACLY and total ACLY levels for hepatic alterations and overweight, we next focused to determine transcriptional changes and molecular pathways altered by ACLY inhibitors (HC, SB, and Bemp) in siRNA ACLY interfered and siRNA control AML12 hepatocytes (Figure S2A–L). We used ingenuity pathway analysis (IPA), gene set enrichment analysis (GSEA), ShinyGO, and Metascape platforms for transcriptional analyses. Interestingly, Metascape and ShinyGO analysis showed that siRNA ACLY interference produced a profound modulation of the transcriptome, resulting in alterations in pathways related to extracellular matrix reorganization (Naba matrisome associated), monocarboxylic acid metabolism, and response to xenobiotics (Figure S2M,N). Noticeably, alterations in the hepatic control of coagulation processes were also highlighted, in accordance with the association of total ACLY levels in human liver biopsies (Figure 1e–g and Figure S2N). The analysis of the effects of ACLY inhibitors in AML12 cells (siRNA control) indicated that each inhibitor modulates differently the transcriptional profile, although a subset of genes were commonly altered by HC, SB, and Bemp (Figure S2O). Alteration of the transcriptional profiles was also observed in siRNA ACLY interfered cells treated with ACLY inhibitors, indicating that HC, SB, and Bemp produce certain transcriptional modulations in the absence of ACLY expression (Figure S2P). The specific analysis of the effects of HC, SB, and Bemp in control (siRNA control) and siRNA ACLY interfered AML12 cells showed that each inhibitor alters differentially the transcriptional profile if ACLY expression is blocked (Figure S2Q–S). Supporting the role of HC, SB, and Bemp as ACLY inhibitors, IPA analysis indicated that pathways related to cholesterol synthesis, Ac‐CoA synthesis and immunomodulatory processes were differentially altered in cells expressing ACLY (siRNA control) treated with the different ACLY inhibitors when compared to siRNA ACLY interfered cells treated with HC, SB, or Bemp (Figure S2T–V) (Baardman et al., 2020; Lauterbach et al., 2019; Sola‐García et al., 2023). The specific analysis of the effects of HC, SB, and Bemp by GSEA in ACLY‐expressing cells (siRNA control) indicated that ACLY inhibitors modulate pathways related to immunomodulation, mTor signaling, and cholesterol/fatty acid metabolism (Figure 2a–c).

Based on the modulations induced by ACLY inhibitors in lipid metabolism, we further analyzed alterations in lipid content in AML12 hepatocytes siRNA interfered for ACLY expression treated or not with the ACLY inhibitors HC, SB, and Bemp. siRNA ACLY interfered AML12 hepatocytes showed a trend toward increased lipid content that did not reach statistical significance. Treatment with the different ACLY inhibitors (HC, SB, and Bemp) produced a marked increase in lipid content in siRNA control cells that was not further increased in siRNA ACLY interfered cells (Figure 2d). The metabolic activity of AML12 cells determined by MTT test, which can contribute to accumulate lipids if restricted, was reduced in siRNA ACLY interfered cells (Figure 2e). These effects were mimicked by the specific ACLY inhibitors HC and SB. However, the MTT activity of AML12 cells treated with Bemp, an ACLY inhibitor that also has an Ampk activatory action, failed to reach statistical significance (Pinkosky et al., 2013). We further analyzed whether mitochondrial functional dynamics were altered in siRNA ACLY interfered AML12 cells treated or not with ACLY inhibitors. The analysis of untreated siRNA ACLY interfered cells indicated that slight effects were produced in oxygen consumption rate (OCR) and extracellular acidification rate (ECAR) when compared to untreated siRNA control cells (Figure 2f–k). Remarkably, SB and Bemp reduced FCCP‐induced maximal OCR moderately, while HC exhibited a milder reduction on OCR that did not reach statistical significance in the majority of the time points in siRNA control cells (Figure 2f–h). ECAR was found specifically reduced in siRNA control cells treated with Bemp, suggesting a potent inhibition of glycolysis (Figure 2i–k). Differences promoted by HC and SB were still observable in siRNA ACLY interfered cells, while differences promoted by Bemp were not found in siRNA ACLY interfered cells, which suggests that certain effects of ACLY inhibitors do not require ACLY expression. Overall, these results indicate that diverse widely‐used ACLY inhibitors have common and specific effects in AML12 cells, and highlight that, among the ACLY inhibitors tested, HC produces the mildest effects in mitochondrial performance.

### 2.3 Hydroxycitrate delays early mortality in mice

**FIGURE 3:**
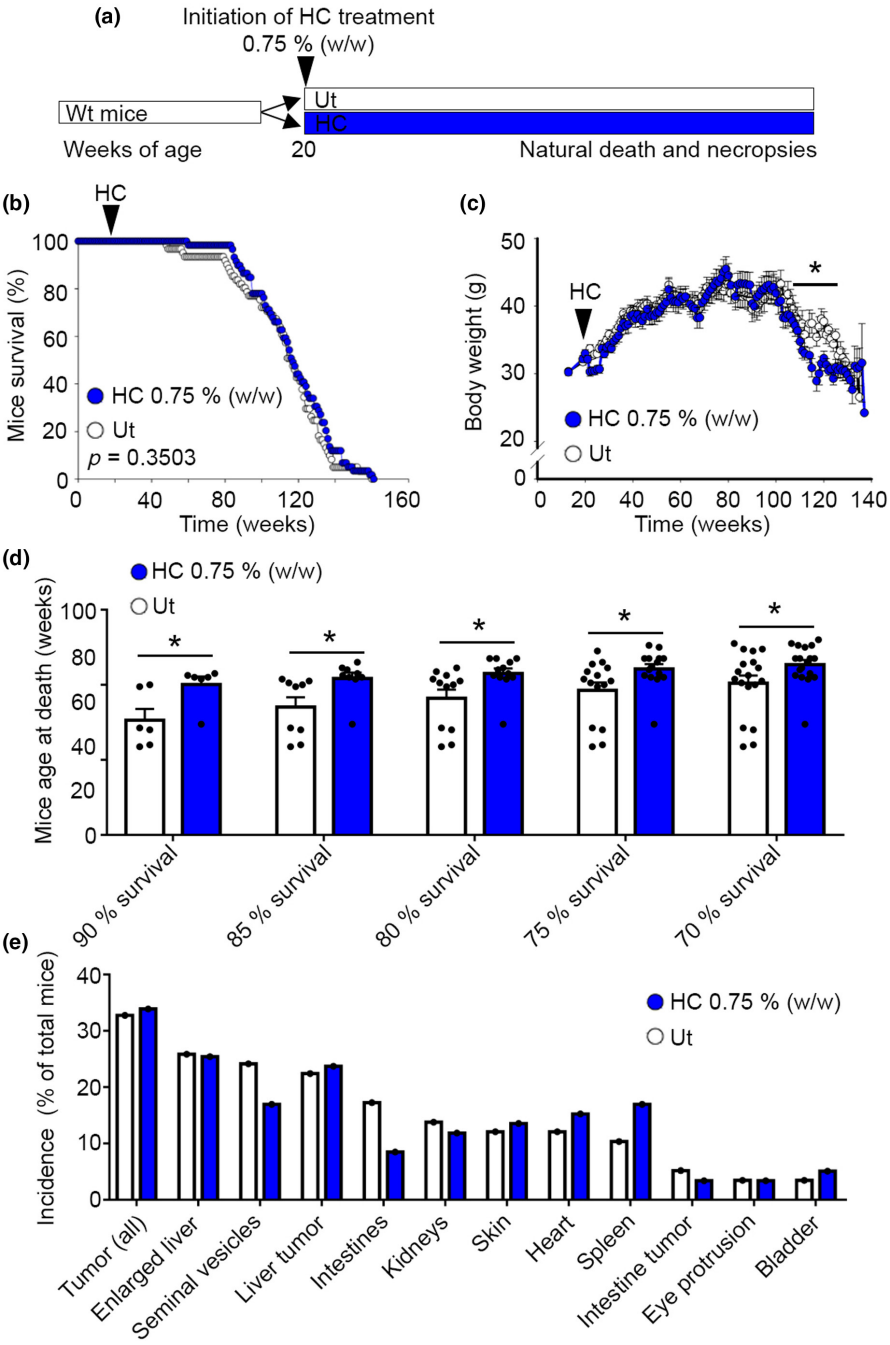
The ACLY inhibitor HC delays early mortality in mice. (a) Graphical representation of the experimental model. (b) Kaplan–Meier survival curve of wild‐type mice treated or not with 0.75% (w/w) HC. *n* = 61 Ut, *n* = 59 HC. Survival by logrank. (c) Body weight during longevity assay. *n* = 24 Ut, *n* = 20 HC at the initiation of the study. Student's *t*‐test. (d) Age at death of the shortest living mice in longevity study. *n* = 6 for 90% survival (i.e., age of death of the 10% shortest living mice). *n* = 9 for 85% survival. *n* = 12 for 80% survival. *n* = 15 for 75% survival. *n* = 18 for 70% survival. Student's *t*‐test. (e) Major pathologies detected at time of death. *n* = 58 Ut, *n* = 59 HC. Fisher exact test. HC, hydroxycitrate; r.u., relative units; Ut, untreated. Data shown are the means ± SEM or incidence over total mouse population. *p < 0.05 HC vs. Ut.

Given the important role of ACLY in organismal health, we set out to investigate whether chronic treatment with ACLY inhibitors can extend the lifespan of wild‐type mice fed with a heathy standard diet (STD). For that, we treated adult wild‐type C57BL/6 male mice with the natural ACLY inhibitor HC that had the mildest effects in mitochondrial function. Our results indicated the lack of significant alterations in global longevity and reduced body weight late in life (i.e., Weeks 115–125 of life) in the absence of differences in energy intake (Figure 3a–c and Figure S3A). However, mice treated with HC exhibited significant delays in spontaneous early mortality at 70%–90% mice survival in the longevity study (Figure 3d). Remarkably, mice treated with HC showed similar incidence of major pathologies at time of death, suggesting that chronic exposure to HC does not produce toxicity at the given dose (Figure 3e). Overall, these data indicate that HC could alter molecular mechanisms of aging that predispose to early mortality in mammals.

### 2.4 Hydroxycitrate does not improve glucoregulation and alters locomotor function in healthy‐fed mice

HC is a known competitive inhibitor of the ACLY, a protein at the interphase of lipid, carbohydrate, and protein metabolism. Therefore, it is tempting to speculate that delay in early mortality could be due to alterations in metabolic health, which is known to predispose to early death (Mitchell et al., 2014). Mice in the longevity assay were evaluated for indirect calorimetry to determine spontaneous changes in activity, energy expenditure, and to evaluate preferences in lipid and carbohydrate utilization. Results indicated that HC does not alter the aforementioned parameters in mice (Figure S4A–G). We next focused to determine effects of HC in glucoregulation in wild‐type mice treated with HC fed with a healthy STD or high‐fat diet (HFD). Our results determined that mice treated with HC fed with a healthy STD did not exhibit alterations in glucoregulation and insulinemia in oral glucose tolerance test (OGTT), intraperitoneal pyruvate tolerance test (IPPTT), and insulin tolerance test (ITT) (Figure S4H–K). Mice only exhibited a modest increase in fasting circulating glucose on mice fed a healthy STD that did not contribute to increase the homeostatic assessment of insulin resistance (HOMA‐IR), while aspartate transaminase (AST) and ALT markers of liver damage were not altered (Figure S4L–P). Moreover, mice exhibited a trend toward increased glycated hemoglobin levels, suggesting long‐term effects in glycemic levels in STD‐fed mice treated with HC (Figure S4Q). Separated experiments showed a nearly significant (*p* = 0.06) reduced fasting‐induced energy intake, supporting seminal observations indicating the appetite suppressant activity of HC (Figure S4R) (Sullivan et al., 1974; Westerterp‐Plantenga & Kovacs, 2002). We next focused to determine the effects of HC in other relevant aspects of aging, such as neurocognitive health and muscle strength. Neurocognitive test indicated the lack of effects of HC in spatial memory (Barnes Maze test) (Figure S4S–U). Fear conditioning and performance on novel object recognition test were also unaffected in HC‐treated mice fed with STD, indicating the lack of effects of HC treatment in neurocognitive health (Figure S4V–X). Remarkably, HC produced an increase in wire hang performance, which reflects preferentially fatigability, while reduced rotarod performance (motor coordination) in mice fed with a healthy STD (Figure S4Y,Z). These results suggest the presence of alterations in muscle tissue in mice fed with a standard healthy diet treated with HC.

Mice fed with a HFD treated with HC exhibited similar body weight, energy intake, as well as glucose and pyruvate tolerance when compared to untreated HFD‐fed mice (Figure S5A–F). HFD‐HC mice exhibited enhanced insulin tolerance when compared to HFD mice (Figure S5G). Similar to STD‐fed mice, mice treated with HC in a HFD exhibited a trend toward increased fasting glucose that did not lead to alterations in the HOMA‐IR index (Figure S5H–J). Interestingly, fasting‐induced energy intake was unaltered in these mice, while glycated hemoglobin levels were significantly reduced, indicating that, under metabolically challenging conditions such as HFD, HC reduces long‐term glycemic levels (Figure S5K,L). HC did not produce remarkable effects in neurocognitive health and muscle strength in HFD‐fed mice (Figure S5M–Q). These results show that only in metabolically challenging conditions (i.e., HFD), HC alters glucoregulatory processes, indicating that, in healthy feeding regimens, the effects on longevity are separated from improvements in metabolic health.

### 2.5 HC promotes a rich energetic status in the liver

**FIGURE 4:**
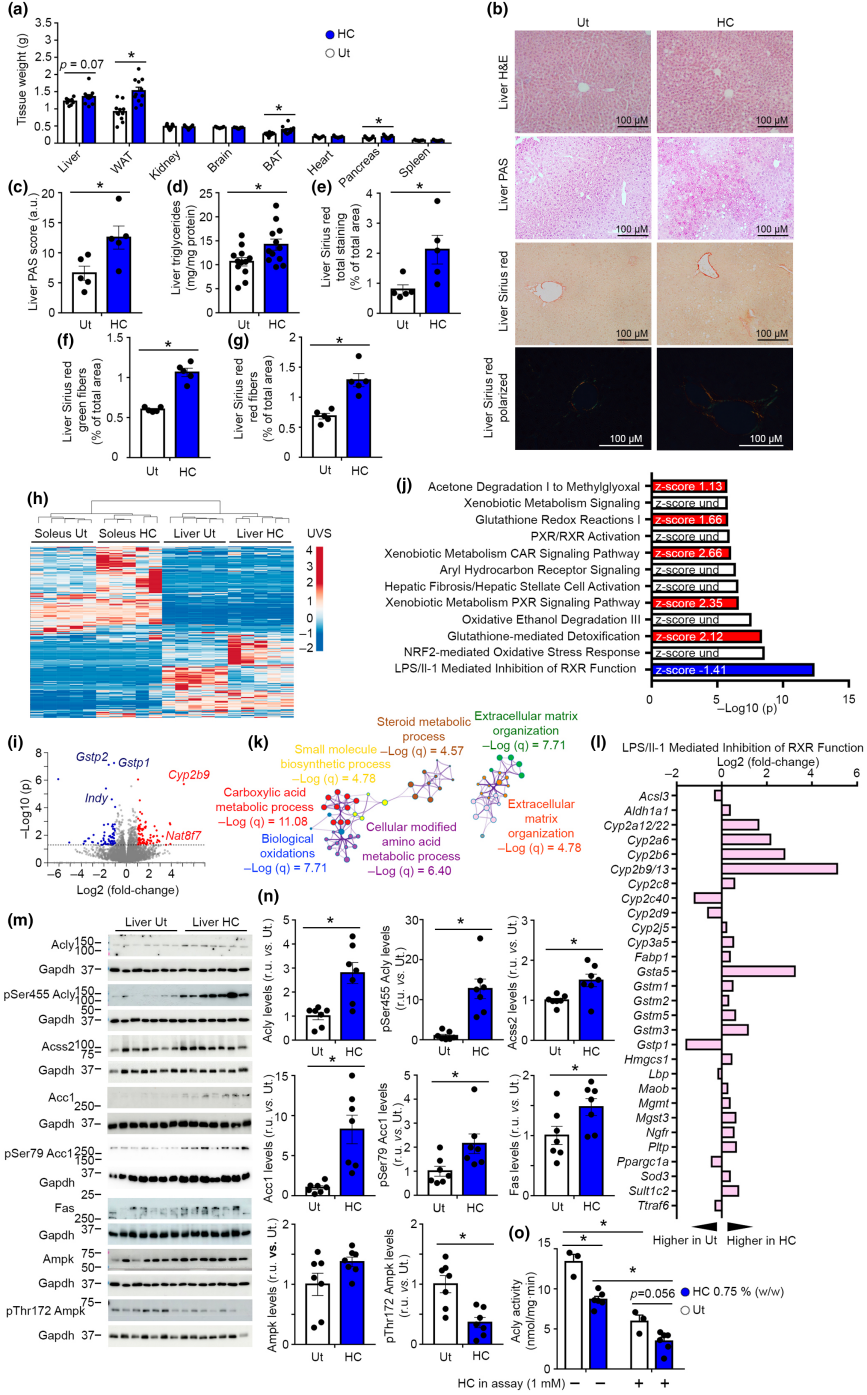
HC increases glycogen and lipid accumulation in the liver. (a) Tissue weight at euthanization in mice fed with healthy STD at 41 weeks of age (21 weeks of treatment). *n* = 11. Student's *t*‐test. (b) Histological analysis of liver tissue to determine the tissue structure by hematoxylin and eosin staining, glycogen depositions by PAS staining and collagen depositions by Sirius red staining. *n* = 5. (c) PAS score in liver tissue; *n* = 5; Student's *t*‐test. (d) Triglyceride content in liver tissue; *n* = 12; Student's *t*‐test. (e) Sirius red total area in liver tissue; *n* = 5; Student's *t*‐test. (f) Green fibers under polarized light in Sirius red staining in liver tissue; *n* = 5; Student's *t*‐test. (g) Red fibers under polarized light in Sirius red staining in liver tissue; *n* = 5; Student's *t*‐test. (h) Heatmap of raw counts of differentially expressed genes (*p* ≤ 0.05, Log2 of fold‐change ≥1 or ≤−1) in soleus and liver tissue; *n* = 5. (i) Volcano plot depicting the fold change and the statistical significance of transcripts in liver. Over‐expressed transcripts are highlighted in red and downregulated transcripts are highlighted in blue. Reference line shows the threshold of significance. (j) 12 top significantly modulated canonical pathways by ingenuity pathway analysis in liver. Bars with positive *z*‐scores are filled in red and bars with negative *z*‐scores are filled with blue. *n* = 5. (k) Top 7 enriched terms by Metascape analysis in liver tissue. *n* = 5. (l) Significantly modulated genes in the canonical pathway “LPS/Il‐1‐mediated inhibition of RXR function” in liver tissue. *n* = 5. (m) Representative western blots of hepatic proteins of mice treated with HC. *n* = 7. (n) Densitometric quantification of western blots shown in panel m; *n* = 7; Student's *t*‐test. (o) Hepatic ACLY activity. ACLY activity was determined in the presence or not of HC in the assay; *n* = 3 Ut, *n* = 6 HC. Two‐way ANOVA plus Tukey post hoc test. a.u., arbitrary units; H&E, hematoxylin and eosin; HC, hydroxycitrate; r.u., relative units; Und, undetermined; Ut, untreated; UVS, unit variance scaling. Data shown are the means ± SEM. *p < 0.05 HC vs. Ut.

We next focused to analyze the tissues of mice treated with HC for 21 weeks (41 weeks of age). At the time of euthanization, the weight of white adipose tissue (WAT), brown adipose tissue (BAT), and pancreas were greater in mice treated with HC fed with a healthy STD, while alterations in tissue weight were not observed in HFD‐fed mice treated with HC (Figure 4a and Figure S6A). Noticeably, hepatic tissue of healthy‐fed mice treated with HC exhibited enhanced glycogen and triglyceride accumulation (Figure 4b–d). This is particularly interesting, as ACLY, the target of HC, participates in de novo lipogenesis. In addition, Sirius red staining showed greater collagen depositions in mice treated with HC (Figure 4b,e–g). These results agree with the transcriptional profile of AML12 cells treated with ACLY inhibitors (Figure 2 and Figure S2).

Alterations in lipid profiles have been associated with longevity, indicating that a higher ratio of monounsaturated fatty acids to polyunsaturated fatty acids predisposes to longer lifespan (Puca et al., 2008; Schroeder & Brunet, 2015). The analysis of lipid species indicated major alterations in the lipidome in mice treated with HC fed with STD (Figure S6B–D). The overall pattern of fatty acid methyl esters (FAMES) in the liver of STD‐fed mice treated with HC was associated with decreases in stearic acid (saturated), increases in monounsaturated species (16:1 and 18:1) and decreases in arachidonic acid (20:4) (Figure S6B). Interestingly, no major changes were observed in the WAT of STD‐fed mice treated with HC (Figure S6B). The analysis of diglyceride and triglyceride species of STD‐fed mice treated with HC showed an increase in shorter species rich in unsaturations (50:4) and reductions in longer species rich in unsaturations (54:4 and 56:8) (Figure S6C,D). In the WAT, we found minimal alteration in diglycerides species, increases in shorter triglycerides (50:1, 50:3/52:4, and 52:6) and reduced levels of longer triglycerides (54:2, 54:3, and 54:4) (Figure S6C,D). The analysis of hepatic FAMES in mice fed with HFD indicated the absence of major changes, with a slight increase of palmitic acid (16:0) (Figure S6E). Overall, lipid analyses reflect that, in mice fed with healthy STD, HC produces lipidic changes in the liver that can contribute to modulate membrane fluidity and signaling events relevant to sustain metabolic health and survival.

We next focused to determine transcriptional modulations that could drive toward delayed early mortality in mice treated with HC. Transcriptional analysis of the liver tissue indicated a robust modulation of the transcriptome (Figure 4h,i). In the liver, 156 genes were significantly altered (*p* ≤ 0.05, Log2 of fold‐change ≥1 or ≤−1). Among those genes, there was a downregulation of several glutathione S‐transferases pi (*Gstp1* and *Gstp2*). Moreover, the liver of HC‐treated mice exhibited reduced mRNA levels of *Indy*, a gene coding for the plasma membrane citrate transporter, which reduced expression is known to resemble a calorie restriction phenotype including protection from metabolic deregulations (Birkenfeld et al., 2011). Transcriptional profiles of liver supported the modulations of pathways related to citrate metabolism (i.e., *Indy*). We further analyzed the molecular pathways altered by HC using IPA, GSEA, ShinyGO, and Metascape platforms for transcriptional analyses. GSEA indicated that unfolded protein response was downregulated, while beta cell signaling, which refers to alterations in expression levels of transcripts involved in glucose metabolism such as *Hnf1a*, *Hnf‐3b*, *Gck*, and *Pklr*, was upregulated (i.e., positive enrichment score) (Figure S6F). Analyses using IPA, ShinyGO, and Metascape indicated a profound alteration in pathways related to extracellular matrix, collagen depositions, responses to xenobiotics, and fatty acid metabolism (Figure 4j,k and Figure S6G). Among those alterations, IPA indicated that the main pathways altered by HC in hepatic tissue included LPS‐Il1‐mediated inhibition of retinoid X receptors (RXR) function, NRF2‐mediated oxidative stress responses, glutathione‐mediated detoxification and pathways related to xenobiotic metabolism. In this line, expression levels of significantly altered transcripts in LPS‐Il1‐mediated inhibition of RXR function showed increased RNA expression of several metabolizing enzymes (Cyp family) and genes involved in glutathione metabolism (Gst family) (Figure 4l). These pathways are suggestive of effects derived from the chronic exposure to the xenobiotic (i.e., HC) in hepatic tissue.

We next focused the analysis of liver tissue to determine the modulation of molecular mechanisms in which ACLY plays an important role, such as histone acetylation and energy metabolism. Epigenetic modulation of histones via acetylation is known to occur upon restriction of ACLY expression in cell cultures (Wellen et al., 2009). Our experiments indicated the absence of global alterations in histone and non‐histone protein acetylation in the liver of mice treated with HC, which is in agreement with previous work evaluating the effects of the ACLY inhibitor SB in mouse liver (Figure S6H–K) (Sola‐García et al., 2023). Interestingly, despite the fact that HC is a well‐established inhibitor of the ACLY, protein expression levels of ACLY and its phospho‐active pSer455 isoform were found to be increased in the liver of HC‐treated mice (Figure 4m,n). However, ACLY activity was reduced in liver lysates of mice treated with HC (Figure 4o). Additional experiments showed that HC administration in the enzymatic assay reduced ACLY activity in the liver of Ut‐ and HC‐treated mice, indicating effective inhibition of ACLY by HC. These results suggest that as a compensatory mechanism, upon HC‐mediated pharmacological ACLY inhibition, the transcriptional and posttranscriptional control of ACLY activity is enhanced. We found greater expression levels of cytosolic Acyl‐coenzyme A synthetase short‐chain family member 2 (Acss2), which participates in an alternative pathway that generates cytosolic Ac‐CoA, in the livers of mice treated with HC (Figure 4m,n). These results are in agreement with several studies showing enhanced Acss2 expression upon restriction of ACLY expression (Martinez Calejman et al., 2020; Wellen et al., 2009). Remarkably, phospho‐active pThr172 Ampk levels were found reduced, indicating a rich energetic status, while total levels of Acc1, the phospho‐inactive pSer79 Acc1, and Fas were increased in the livers of HC‐treated mice (Figure 4m,n). pSer235/236 S6, pThr37/46 4E‐bp1, and pSer473 Akt, markers of mTOR and Akt activity, respectively, were unaltered, suggesting that these pathways do not contribute substantially to the phenotype observed (Figure S6L,M). Restricted Ampk phosphorylation concomitant with enhanced ACLY phosphorylation and increased hepatic triglyceride levels suggest alterations in de novo lipogenesis or lipid expenditure (i.e., mitochondrial metabolism), as found in AML12 cells treated with ACLY inhibitors (Figure 2).

### 2.6 HC influences pathways critical for tissue regeneration in skeletal muscle

**FIGURE 5:**
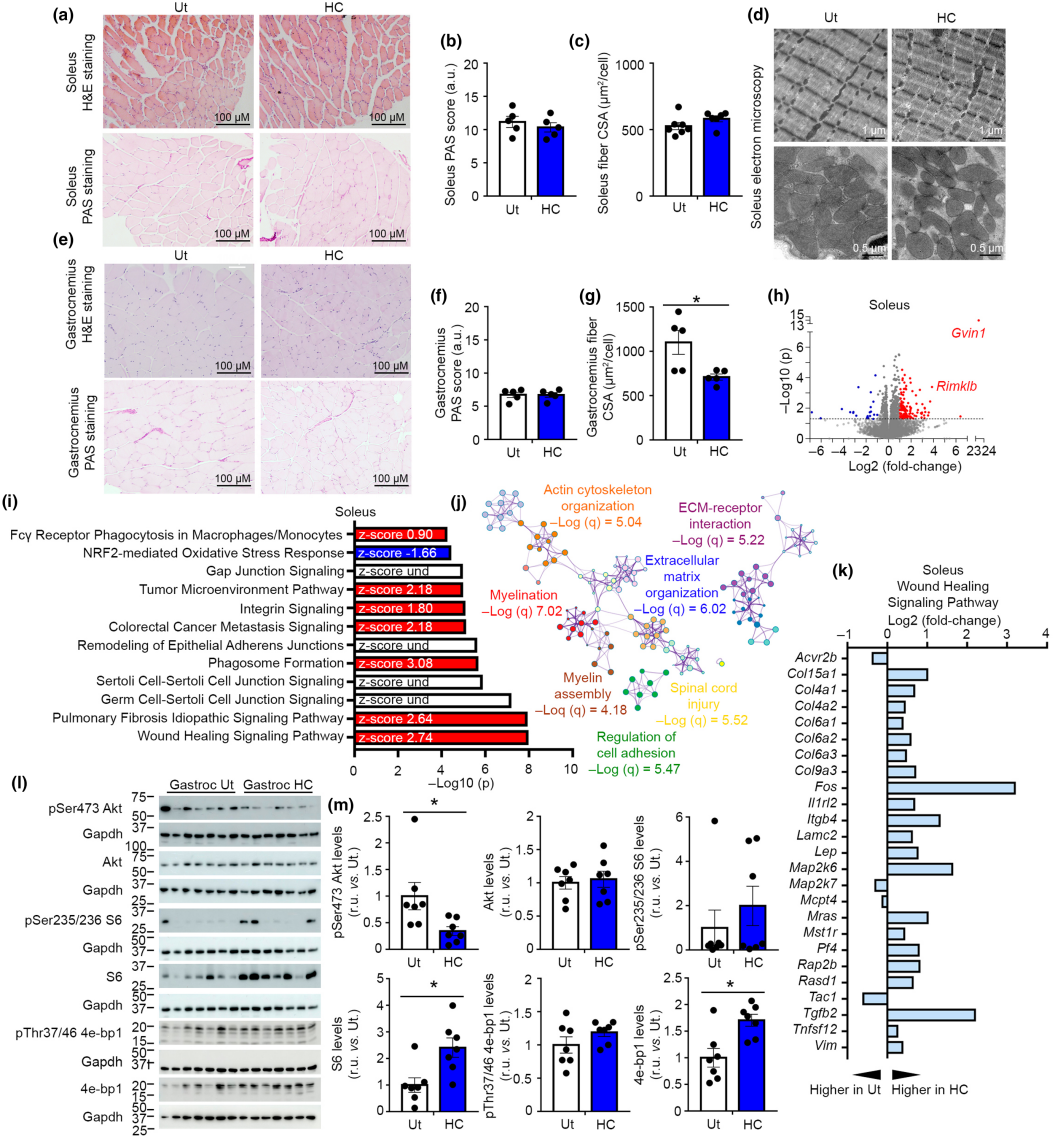
HC modulates pathways involved in tissue regeneration in skeletal muscle. (a) Histological analysis of cellular structure by hematoxylin and eosin staining and glycogen depositions by PAS staining in soleus tissue; *n* = 5. (b) PAS score in soleus tissue; *n* = 5; Student's *t*‐test. (c) Soleus fiber CSA; *n* = 7 Ut, *n* = 6 HC; Student's *t*‐test. 150–274 fibers were counted per soleus. (d) Ultrastructural analysis of the soleus by electron microscopy; *n* = 4. (e) Histological analysis of cellular structure by hematoxylin and eosin staining and glycogen depositions by PAS staining in gastrocnemius; *n* = 5. (f) PAS score in gastrocnemius tissue; *n* = 5; Student's *t*‐test. (g) Gastrocnemius fiber CSA; *n* = 5; Student's *t*‐test. 477–2261 fibers were counted per gastrocnemius. (h) Volcano plot depicting the fold change and the statistical significance of transcripts in soleus. Over‐expressed transcripts are highlighted in red and downregulated transcripts are highlighted in blue. Reference line shows the threshold of significance; *n* = 5. (i) 12 top significantly modulated canonical pathways by Ingenuity Pathway Analysis in soleus. Bars with positive *z*‐scores are filled in red and bars with negative *z*‐scores are filled with blue. *n* = 5. (j) Top 7 enriched terms by Metascape analysis in the soleus. *n* = 5. (k) Significantly modulated genes in the canonical pathway “wound healing signaling pathway” in the soleus; *n* = 5. (l) Representative western blots of gastrocnemius mice treated with HC fed with healthy STD at 41 weeks of age (21 weeks of treatment); *n* = 7. (m) Densitometric quantification of western blots shown in panel l; *n* = 7. Student's *t*‐test. a.u., arbitrary units; ECM, extracellular matrix; Gastroc, gastrocnemius; H&E, hematoxylin and eosin; HC, hydroxycitrate; r.u., relative units; Und, undetermined; Ut, untreated. Data shown are the means ± SEM. *p < 0.05 HC vs. Ut.

Given the alterations in locomotor function, we analyzed the effects of HC in skeletal muscle. The analysis in the soleus of cell size, glycogen content by Periodic acid–Schiff (PAS) staining and the fiber types (i.e., Myh7‐positive fibers) did not reveal major differences in this slow‐twitch muscle type (Figure 5a–c and Figure S7A). In addition, the ultrastructure analysis of soleus by electron microscopy did not evidence morphological changes in the mitochondria of HC‐treated mice, which showed similar circularity, matrix electron density, and cristae development when compared to untreated mice (Figure 5d and Figure S7B–D). In the gastrocnemius, we found similar glycogen content and reduced cellular cross‐sectional area (CSA) in mice treated with HC (Figure 5e–g). Further analyses of slow switch Myh7‐expressing fibers indicated the absence of differences in the gastrocnemius (Figure S5E–G). Similar to the liver, FAMES in the gastrocnemius of STD‐fed mice treated with HC was associated with decreases in short saturated acid species (16:0 and 18:0), increases in their monounsaturated species (16:1 and 18:1) (Figure S6B). The analysis of diglyceride and triglyceride species in the gastrocnemius determined reduced levels of 54:2 and 54:3, which were concomitant to increased 54:6 levels, suggesting greater levels of unsaturations in fatty acids forming diglycerides and triglycerides (Figure S6C,D). Overall, hepatic and gastrocnemius lipid analyses indicated similar changes promoted by HC.

Transcriptional analysis of soleus indicated a robust modulation of the transcriptome with a distinctive transcriptional profile when compared with liver tissue in mice treated with HC (Figure 4h). In the soleus, 143 transcripts were significantly altered, with a remarkable high proportion of over‐expressed genes (~85%) (Figure 5h). Among over‐expressed genes, we found *Gvin1*, an interferon inducible pseudogene, and *Rimklb*, a gene with predicted citrate‐l‐glutamate ligase activity. In the soleus GSEA analysis showed marked increases in processes related to inflammation and core metabolic processes (Figure S7H). Further analyses using IPA, ShinyGO, and Metascape indicated a profound alteration in myelination, extracellular matrix reorganization, and oxidative stress responses (Figure 5i,j and Figure S7I). IPA analysis on the soleus of HC‐treated mice exhibited the strongest modulation on the wound healing canonical pathway, which involved the upregulation of several collagens and Tgfb2 (Figure 5i,k). Transcriptomic analysis of soleus tissue indicated modulations in regenerative pathways, a process that requires the activation of anabolic pathways (Squarize et al., 2010). However, the inhibition of these pathways have been normally associated with longer lifespan (Harrison et al., 2009). We evaluated expression levels of ACLY, as well as markers of energy status and mitochondrial content in gastrocnemius lysates of mice treated with HC. As expected, ACLY expression in gastrocnemius was reduced when compared to hepatic expression (Beigneux et al., 2004), and HC did not alter ACLY expression in this muscle (Figure S7J,K). Data also indicated that markers of mitochondrial biogenesis and content were similar in mice treated with HC when compared to untreated mice (Figure S7L,M). pThr172 Ampk levels were unaltered in mice treated with HC, although downstream Ampk signaling (pSer72 Acc) was reduced (Figure S7L,M). Further analyses of gastrocnemius tissue of mice treated with HC showed reduced Akt signaling, suggesting reduced anabolic signaling (Figure 5l,m). Remarkably, phosphorylated isoforms of several mTOR targets, such as S6 and 4E‐bp1, were found to be unaltered in mice treated with HC, although total levels of these markers were higher in the same samples (Figure 5l,m). These data indicate that expression levels of anabolic pathways are increased in the muscles of mice treated with HC, although signaling events sustain similar, or even reduced, activity in mice treated with HC.

### 2.7 HC potentiates muscle regeneration in vivo

**FIGURE 6:**
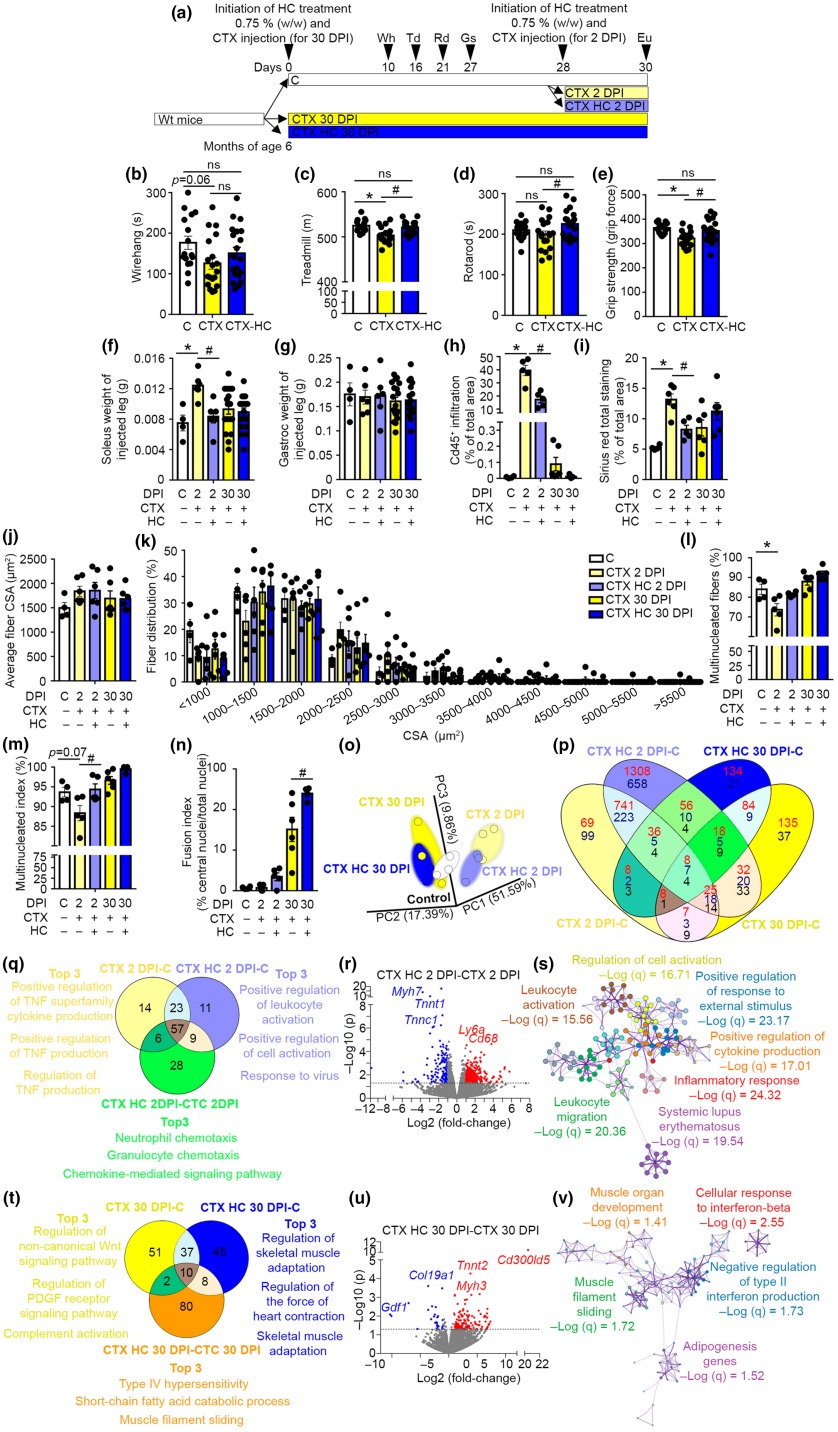
HC potentiates muscle regeneration in vivo. (a) Graphical representation of the experimental model. 6 ± 1‐month‐old Wild‐type male C57BL/6 mice were injected with CTX to generate muscle damage. Then, HC treatment was initiated and physical strength was evaluated using different test. At Day 2 or Day 30 post CTX injection, mice were euthanized and tissues were weighed and harvested. Eu, euthanization; Gs, grip strength; Rd, rotarod; Td, treadmill; Wh, wire hang. (b) Wire hang performance at 10 DPI. *n* = 16 C, *n* = 19 CTX, *n* = 20 CTX‐HC. One‐way ANOVA plus Tukey post hoc test. (c) Treadmill performance at 16 DPI. *n* = 16 C, *n* = 18 CTX, *n* = 20 CTX‐HC. One‐way ANOVA plus Tukey post hoc test. (d) Rotarod performance at 21 DPI; *n* = 16 C, *n* = 19 CTX, *n* = 20 CTX‐HC. One‐way ANOVA plus Tukey post hoc test. (e) Grip strength performance at 27 DPI; *n* = 15 C, *n* = 19 CTX, *n* = 20 CTX‐HC. One‐way ANOVA plus Tukey post hoc test. (f) Soleus weight of injected leg; *n* = 4 C, *n* = 6 CTX 2 DPI, *n* = 6 CTX‐HC 2 DPI, *n* = 19 CTX 30 DPI, *n* = 18 CTX‐HC 30 DPI. One‐way ANOVA plus Tukey post hoc test. (g) Gastrocnemius weight of injected leg; *n* = 4 C, *n* = 6 CTX 2 DPI, *n* = 6 CTX‐HC 2 DPI, *n* = 19 CTX 30 DPI, *n* = 17 CTX‐HC 30 DPI. One‐way ANOVA plus Tukey post hoc test. (h) Cd45^+^ immune infiltration total area in soleus; *n* = 4 C, *n* = 5 CTX 2 DPI, *n* = 5 CTX‐HC 2 DPI, *n* = 6 CTX 30 DPI, *n* = 6 CTX‐HC 30 DPI. One‐way ANOVA plus Tukey post hoc test. (i) Sirius red total area in soleus; *n* = 4 C, *n* = 6 CTX 2 DPI, *n* = 5 CTX‐HC 2 DPI, *n* = 6 CTX 30 DPI, *n* = 6 CTX‐HC 30 DPI. One‐way ANOVA plus Tukey post hoc test. (j) Fiber CSA in soleus; *n* = 4 C, *n* = 6 CTX 2 DPI, *n* = 6 CTX‐HC 2 DPI, *n* = 6 CTX 30 DPI, *n* = 6 CTX‐HC 30 DPI. One‐way ANOVA plus Tukey post hoc test. (k) Fiber CSA distribution in soleus; *n* = 4 C, *n* = 6 CTX 2 DPI, *n* = 6 CTX‐HC 2 DPI, *n* = 6 CTX 30 DPI, *n* = 6 CTX‐HC 30 DPI. One‐way ANOVA plus Tukey post hoc test. (l) Multinucleated fibers in soleus; *n* = 4 C, *n* = 5 CTX 2 DPI, *n* = 5 CTX‐HC 2 DPI, *n* = 6 CTX 30 DPI, *n* = 5 CTX‐HC 30 DPI. One‐way ANOVA plus Tukey post hoc test. (m) Multinucleated index in soleus; *n* = 4 C, *n* = 5 CTX 2 DPI, *n* = 4 CTX‐HC 2 DPI, *n* = 6 CTX 30 DPI, *n* = 5 CTX‐HC 30 DPI. One‐way ANOVA plus Tukey post hoc test. (n) Fusion index in soleus; *n* = 4 C, *n* = 5 CTX 2 DPI, *n* = 5 CTX‐HC 2 DPI, *n* = 6 CTX 30 DPI, *n* = 4 CTX‐HC 30 DPI. One‐way ANOVA plus Tukey post hoc test. (o) PCA was applied for transcriptomic analysis in gastrocnemius muscle. Each point corresponds to the PCA analysis of one mouse sample; *n* = 3. (p) Diagram showing significantly altered transcripts in each CTX‐treated group compared to healthy controls. Upregulation (red), downregulation (blue), and reciprocal regulation (black); *n* = 3. (q) Diagram showing common and specific significantly enriched processes in the gastrocnemius of mice injected or not with CTX at 2 DPI. The top 3 most significant enriched processes specific of each comparison are highlighted; *n* = 3. (r) Volcano plots depicting the fold change and the statistical significance of transcripts in the gastrocnemius in the comparison CTX 2 DPI vs. CTX‐HC 2 DPI. Over‐expressed transcripts are highlighted in red and downregulated transcripts are highlighted in blue. Reference line shows the threshold of significance; *n* = 3. (s) Top 7 enriched terms by Metascape analysis in the gastrocnemius in the comparison CTX‐HC 2 DPI vs. CTX 2 DPI; *n* = 3. (t) Diagram showing common and specific significantly enriched processes in the gastrocnemius of mice injected or not with CTX at 30 DPI. The top 3 most significant enriched processes specific of each comparison are highlighted. *n* = 3. (u) Volcano plots depicting the fold change and the statistical significance of transcripts in the gastrocnemius in the comparison CTX 30 DPI vs. CTX‐HC 30 DPI. Over‐expressed transcripts are highlighted in red and downregulated transcripts are highlighted in blue. Reference line shows the threshold of significance; *n* = 3. (v) Significantly enriched terms by Metascape analysis in the gastrocnemius in CTXHC 30 DPI vs. CTX 30 DPI; *n* = 3. C, healthy control mice not injected with CTX; CTX, cardiotoxin; DPI, days post injury; Gastroc, gastrocnemius; HC, hydroxycitrate; Ut, untreated. Data shown are the means ± SEM. *p < 0.05 CTX vs. C. ^#^ *p* < 0.05 CTX‐HC vs. CTX.

We next reasoned that if HC alters aspects of tissue regeneration, such as inflammation, wound healing, and extracellular matrix reorganization, in skeletal muscles, then HC will potentiate muscle regeneration. We used intramuscular injections of the myonecrotic agent cardiotoxin (CTX) to generate muscle damage in the left hind limb of adult wild‐type mice. Subsequently, mice were treated with HC for 2 or 30 days and euthanized (García‐Prat et al., 2016). Body weight and energy intake was similar in mice treated with CTX (Figure S8A,B). Physical performance was evaluated using treadmill, rotarod, wire hang, and grip strength test during the course of the experimentation. CTX produced significant decreases in treadmill, rotarod, and grip strength performance, while a trend toward reduced performance (*p* = 0.06 in One‐way ANOVA) was found in wire hang test (Figure 6a–e). Results indicated that HC increases treadmill, rotarod and grip strength performance when compared to untreated CTX‐injected mice (Figure 6b–e). The analysis of muscle tissue at time of euthanization indicated that soleus weight was increased early upon CTX injection (2 days post injury, DPI) in untreated mice, while soleus weight remained similar to the soleus weight of healthy controls in mice treated with HC (Figure 6f). The soleus weight of the non‐injected leg (i.e., right leg) remained similar in all experimental groups (Figure S8C). A similar gastrocnemius weight was also found in the injected and non‐injected leg of all mice (Figure 6g and Figure S8D).

At histological level, we observed that irrespective of HC treatment, the soleus of mice exposed to CTX accumulated tissue damage early (2 DPI) upon CTX exposure (Figure S8E). Immune infiltration was evident at 2 DPI, although infiltrated area was reduced in HC‐treated mice (Figure 6h and Figure S8F). Evaluation of collagen fibers by Sirius red staining showed increased collagen deposition in CTX‐injected mice at 2 DPI, whereas mice treated with HC exhibited nearly basal Sirius red staining, indicating that HC reduces collagen deposition at 2 DPI (Figure 6i and Figure S8G). Fiber CSA and proportion of large and small fibers was not altered in any experimental group (Figure 6j,k). Multinucleated index and percentage of multinucleated fibers was reduced in CTX‐injected soleus at 2 DPI, whereas these parameters were similar to healthy mice in animals treated with HC at 2 DPI, indicating early effects of HC treatment (Figure 6l,m). Fusion index was increased at 30 DPI in HC‐treated mice when compared to 30 DPI CTX‐Ut mice, while nuclei number per fiber remained unaffected (Figure 6n and Figure S8H). We further evaluated the effects of HC in C2C12 differentiated myoblasts (Das et al., 2017). HC produced increased expression of *Myod* and *Myh7* late in the differentiation process (6 days in differentiation medium), whereas transient ACLY siRNA interference blocked this effect (Figure S8I–K). In order to further delineate the molecular mechanisms altered by HC, we performed a transcriptomic profile of gastrocnemius muscle to determine early and late responses to CTX and HC treatment. Principal component analysis (PCA) indicated that CTX exerts greater alterations in the global transcriptional profile early upon injection (2 DPI) (Figure 6o). The global analysis of differentially expressed genes further supported that changes at 2 DPI were more profound when compared to 30DPI. Noticeably, HC at 2 DPI produced the modulation of a greater number of genes when compared to untreated mice at 2 DPI, suggesting the modulation of different responses (Figure 6p). The specific analysis of the effects of CTX at 2 DPI in HC and untreated mice showed the existence of specific alterations promoted by CTX (CTX Ut vs. healthy control), HC (CTX HC vs. healthy control), and HC in CTX‐injected mice (CTX HC vs. CTX Ut) (Figure 6q). Among those changes, alterations in tumor necrosis factor (TNF) was unequivocally highlighted in the gastrocnemius of CTX Ut mice, whereas cellular activation was specifically highlighted in HC‐treated mice. Further analysis of the effects of HC in mice exposed to CTX (CTX HC 2 DPI vs. CTX Ut 2 DPI) showed the presence of 779 differentially expressed genes (*p* ≤ 0.05, Log2 of fold‐change ≥1 or ≤−1), which resulted in the alteration of pathways related to immunomodulation, cell activation and migration (Figure 6r,s and Figure S8L). These results agree with an important role of ACLY inhibition in the modulation of immunoregulatory processes (Baardman et al., 2020; Lauterbach et al., 2019). We further analyzed late effects of CTX injection and HC treatment. Interestingly, specific pathways altered in mice exposed to CTX (CTX Ut vs. healthy control) at 30 DPI included Wnt signaling and growth factors, suggesting that early regenerative processes are still active, whereas in mice treated with HC (CTX HC vs. healthy control) at 30 DPI‐specific pathways highlighted processes related to muscle function, reflective of later phases of tissue regeneration (i.e., tissue remodeling) (Figure 6t). Further analysis of the effects of HC in mice exposed to CTX (CTX HC 30 DPI vs. CTX Ut 30 DPI) indicated the presence of 155 differentially expressed genes (*p* ≤ 0.05, Log2 of fold‐change ≥1 or ≤−1), which resulted in the alteration of pathways related to muscle filament sliding and responses to interferon β (Figure 6u,v and Figure S8M). Altogether, our results indicate that HC potentiates skeletal muscle regeneration, resulting in improved locomotor function.

## 3 DISCUSSION

Using liver biopsies, we have shown that increased phospho‐active ACLY levels correlate with important metabolic risk markers in humans, such as obesity and MELD, whereas increased total ACLY levels were rather correlated with negative alterations in the hepatic control of the coagulation system. These data support the use of hepatic ACLY levels as diagnostic markers of these pathological conditions. Moreover, our results show that in addition to the translational control of ACLY expression, the elucidation of the regulation of ACLY phosphorylation is clinically relevant. The correlations indicating that pSer455 ACLY and total ACLY levels are associated with diagnostic markers of human disease, suggest that pharmacological ACLY inhibition could produce relevant physiological consequences in humans.

ACLY inhibitors have been defined as calorie restriction mimetics and are expected to promote healthy aging and increase life expectancy (Hofer et al., 2021). This is in part due to the fact that, in principle, upon ACLY inhibition, a reduction of liponeogenesis can be expected, as ACLY produces malonyl‐coenzyme A, the building block for endogenous lipid production. Moreover, reduced malonyl‐coenzyme A levels upon ACLY inhibition can contribute to generate a concomitant increase in mitochondrial lipid uptake, favored by the restriction of the inhibitory effect of malonyl‐coenzyme A in carnitine palmitoyltrasnferase I. In this line, our findings and the scientific literature do not substantiate enhanced mitochondrial metabolism, including a potential increase in the β‐oxidation of fatty acids, in experimental models lacking normal ACLY activity or expression (Das et al., 2015; Sola‐García et al., 2023). Supporting the role of ACLY inhibition to promote healthy aging, several ACLY inhibitors have been developed and investigated in the pathophysiology of several age‐related diseases, including cancer, hepatic steatosis, hyperlipidemia, and diabetes (Hatzivassiliou et al., 2005; Morrow et al., 2022; Sanjay et al., 2021; Verrelli et al., 2022), and the ACLY inhibitor Bemp has been shown to be safe and effective lowering circulating cholesterol levels in the clinic (Pinkosky et al., 2013; Ray et al., 2019; Ruscica et al., 2022). In this report, we show that the naturally occurring ACLY inhibitor HC specifically delays premature death in wild‐type mice fed with a healthy diet, without having significant effects in overall life expectancy. These data indicate that, despite the body of literature proposing HC as a calorie restriction mimetic, this compound failed to produce effects in the main hallmark of calorie restriction (e.g., extending lifespan). Therefore, our results indicate that HC is not a bona fide calorie restriction mimetic.

Despite these facts, we determined that HC has interesting properties in the modulation of muscle physiology and glucoregulatory processes (mainly in HFD) that occur in the absence of neurocognitive effects. In addition, supporting the correlation of phospho‐active ACLY levels and obesity in humans, HC‐treated mice fed with a healthy diet exhibited reduced body weight late in life. Despite the large body of research investigating the effects of HC and HCA derivatives in body weight, glucose, lipid metabolism, and overall health, there is still no consensus on the effectiveness and safety of these chemicals (Anilkumar et al., 2023; Hofer et al., 2021; Zovi et al., 2022). This might be due to the different sources of HC/HCA used. In the majority of the cases, researchers have used garcinia extracts in which the amount of HC is not properly evaluated. Moreover, other compounds used in the formulation of garcinia extracts might have physiological effects, impeding the proper evaluation of the effects of HC/HCA. In this sense, we used ≥95% pure tripotassium HC. Our results indicate that HC does not produce major alterations in glucoregulation in mice fed with a healthy diet, while certain improvements, such as enhanced insulin tolerance and reduced glycated hemoglobin, are observed in mice fed with an unhealthy HFD. Interestingly, we show that the liver of HC‐treated mice fed with a healthy diet accumulated triglycerides, and three commonly used ACLY inhibitors produced net increases in lipid content in AML12 hepatocytes, an observation that has been previously described in cell cultures and mice treated with ACLY inhibitors and genetically modified animals devoid of ACLY expression (Martinez Calejman et al., 2020; Migita et al., 2014; Wang et al., 2010). Separated reports have shown that compensatory mechanisms might occur in cells and organisms devoid of ACLY activity/expression, leading to normal Ac‐CoA levels, which might involve the increase in the expression of the ACSS2, as we observed in the liver of mice treated with HC, and malonyl decarboxylase (Martinez Calejman et al., 2020). It is tempting to speculate that, despite the fact that ACLY is efficiently inactivated by ACLY inhibitors, alternative mechanisms maintain normal Ac‐CoA levels to feed and even foster hepatic liponeogenesis. Supporting the profound metabolic rewiring produced by ACLY inhibitors (Sola‐García et al., 2023), the liver of HC‐treated mice showed reduced ACLY enzymatic activity concomitant with increased levels of phospho‐active pSer455 ACLY and increased phospho‐inactive pSer79 Acc1, which participate early on in de novo lipogenesis, suggesting adaptive mechanisms to long‐term HC exposure. In this line, the effect of ACLY inhibitors in metabolic activity (e.g., mild reduction in mitochondrial performance) can also contribute to accumulate lipids (Sola‐García et al., 2023). Remarkably, the lipid profile of mice treated with HC showed reduced levels of certain species of saturated and polyunsaturated fatty acids, as well as increases in several species of monosaturated fatty acids. These data indicate that HC reduces the accumulation of highly susceptible to oxidation polyunsaturated fatty acids and rigid saturated fatty acids, while increasing monounsaturated species, which have been associated with increases in life expectancy in several species including humans (Galvan et al., 2015; Puca et al., 2008; Schroeder & Brunet, 2015).

Importantly, HC produced moderated effects in skeletal muscles, potentiating wire hang performance while reducing rotarod performance. At transcriptomic level, we determined that pathways involved in tissue regeneration (e.g., wound healing, inflammation, and extracellular matrix reorganization), which involved the up‐regulation of several collagens and Tgfb2, are significantly modulated in mice treated with HC. The knowledge of the role of ACLY and ACLY inhibitors in skeletal muscle is rather limited. Previous reports have shown that ACLY overexpression increases cardiolipin content and improves mitochondrial function in skeletal muscle in mice (Das et al., 2015), whereas ACLY stimulates myoblast differentiation in vitro (Das et al., 2017). These data support a relevant role of ACLY in the functionality of skeletal muscle.

Our results indicate that HC supplementation after CTX‐induced muscle damage produced improvements in several test targeting muscle function. We also determined early effects of HC supplementation in mice exposed to CTX, involving reduced muscle swelling and fibrosis. At this stage (2 DPI), HC‐treated mice exhibited reduced infiltration of Cd45^+^ cells along with alterations in immunomodulatory processes that participate in muscle regeneration, which agrees with a relevant role of ACLY in inflammation. Supporting the important role of ACLY inhibition in macrophage function, several reports have pointed ACLY as an important target to mitigate in vivo inflammatory complications, such as exposure to lipopolysaccharide and atherosclerosis (Baardman et al., 2020; Lauterbach et al., 2019). Furthermore, late effects of HC (30 DPI), indicate potentiation of tissue remodeling, facilitating earlier functional recovery. Our observations indicate that signaling events controlling anabolic pathways, such as AKT and mTOR targets, are not overactivated in healthy mice treated with HC, but total protein expression levels of several mTOR targets are increased in the gastrocnemius. These data suggest that, if needed (e.g., tissue injury), anabolic pathways could be activated in mice treated with HC to favor faster and efficient functional recovery. The fact that HC modulates muscle function in healthy mice and mice treated with CTX has implications for the understanding of molecular mechanisms involved in muscle regeneration. In conclusion, the identification of the chronic effects of HC and other ACLY inhibitors affecting different aspects of tissue function and organismal health provides a better understanding of aging and age‐related degeneration.

### 3.1 Limitations of the study

While analyses in human samples included male and female subjects, our research using laboratory animals was specifically performed in male mice. This is a major limitation of the study particularly relevant, as sex differences exist in lipid and carbohydrate metabolism (Karastergiou & Fried, 2017), and ACLY contributes to sex‐specific metabolic phenotypes (Fernandez et al., 2019). Therefore, additional work using female mice will be necessary to determine the effects of HC in female mice. Our longevity study has been performed in a healthy standard diet. While long‐term effects of HC supplementation in HFD have been investigated herein, other relevant energy sources such as high sugars have not been included in the current report. It would be particularly interesting to determine effects of ACLY inhibitors in high‐sugar diets, such as fructose supplementation, as ACLY is known to exert important responses mediated by carbohydrate response element‐binding proteins (Fernandez et al., 2019).

## 4 METHODS

See Appendix S2.

## AUTHOR CONTRIBUTIONS

IE, MACM, ASG, RLFS, and AMM performed most of the experiments. MSN performed electron microscopy experiments. EMF, MV and JJS performed determinations of lipid species. DMA and CAC and CPM collected clinical data and analyzed human samples. BRG, FM, VCG and AMM interpreted the data. AMM designed the study, secured funding and wrote the manuscript. All authors discussed the results and commented on the manuscript.

## CONFLICT OF INTEREST STATEMENT

VCG and AMM have filed a patent entitled “hidroxicitrato para la regeneración muscular.”

## Supporting information


Figure S1.

Figure S2.

Figure S3.

Figure S4.

Figure S5.

Figure S6.

Figure S7.

Figure S8.

Appendix S1.

Appendix S2.

## Data Availability

The accession number for the RNA‐Seq gene expression data reported in this paper can be obtained from Gene Expression Omnibus (GSE232837). This paper does not report original code.
