# Supplementary figures and images for: Hydroxycitrate delays early mortality in mice and promotes muscle regeneration while inducing a rich hepatic energetic status
Source: Aging Cell. 2024 May 17;23(9):e14205. doi: 10.1111/acel.14205 (PMC11488303; doi:10.1111/acel.14205)

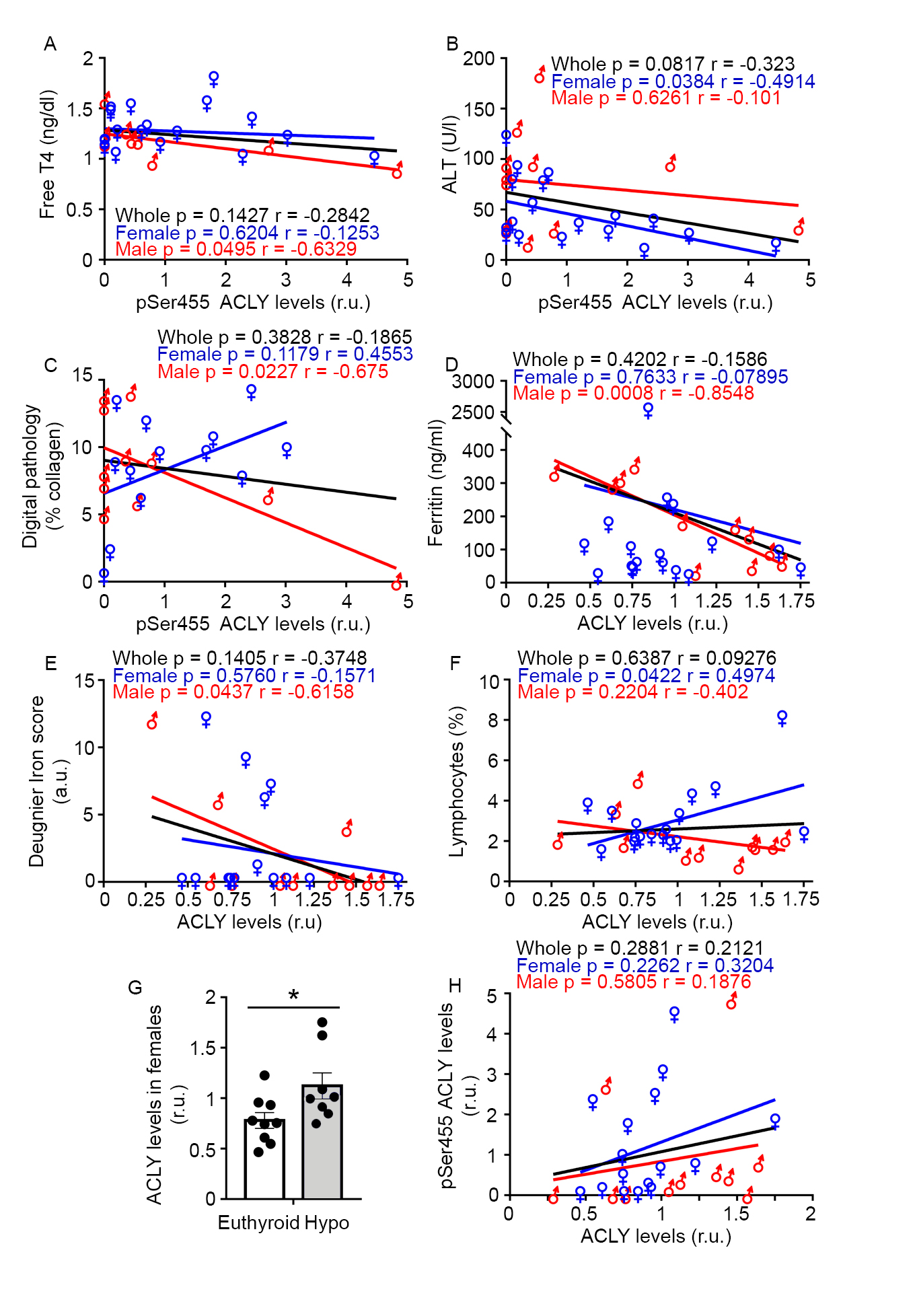

Supplement: Supplementary file 1 — Figure S1. [file ACEL-23-e14205-s006.jpg]

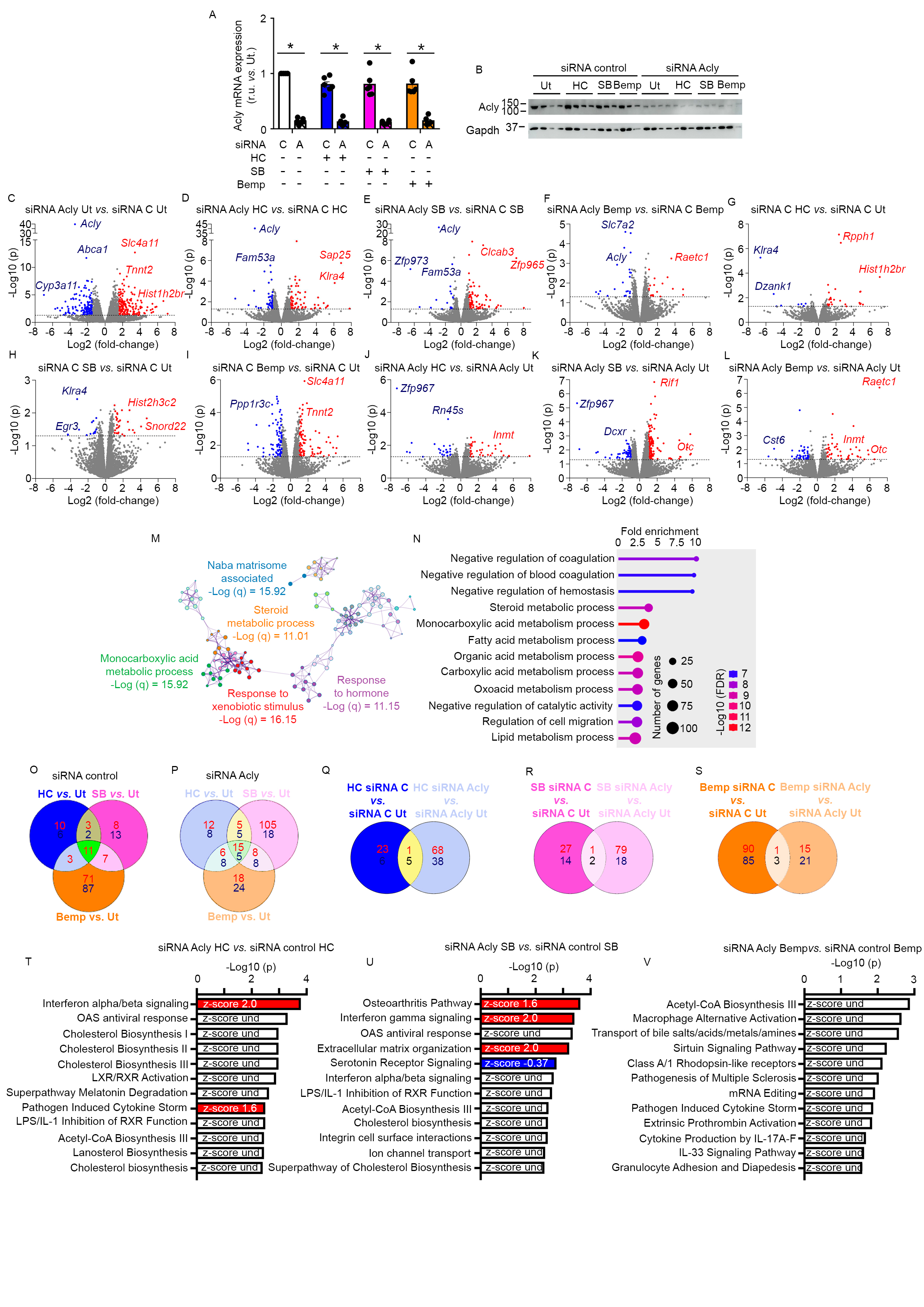

Supplement: Supplementary file 2 — Figure S2. [file ACEL-23-e14205-s005.jpg]

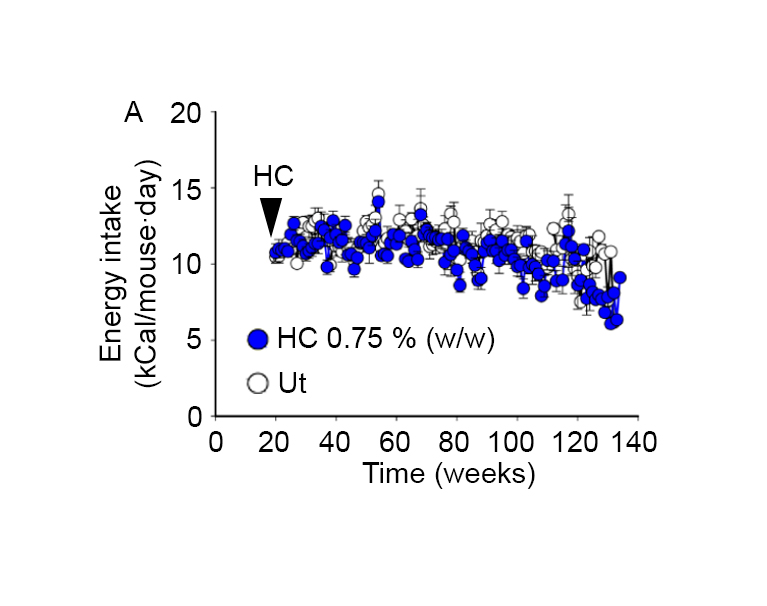

Supplement: Supplementary file 3 — Figure S3. [file ACEL-23-e14205-s002.jpg]

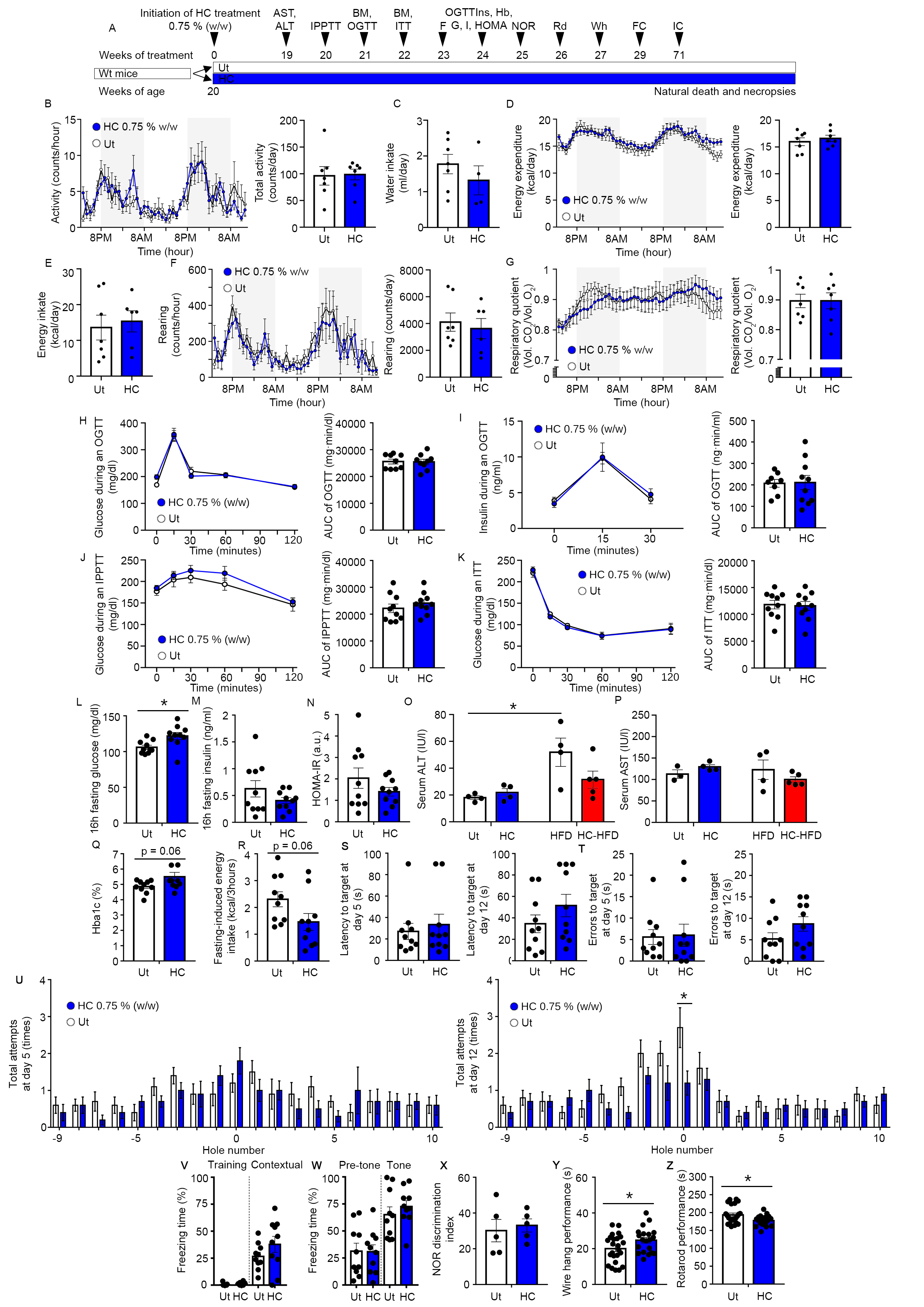

Supplement: Supplementary file 4 — Figure S4. [file ACEL-23-e14205-s004.jpg]

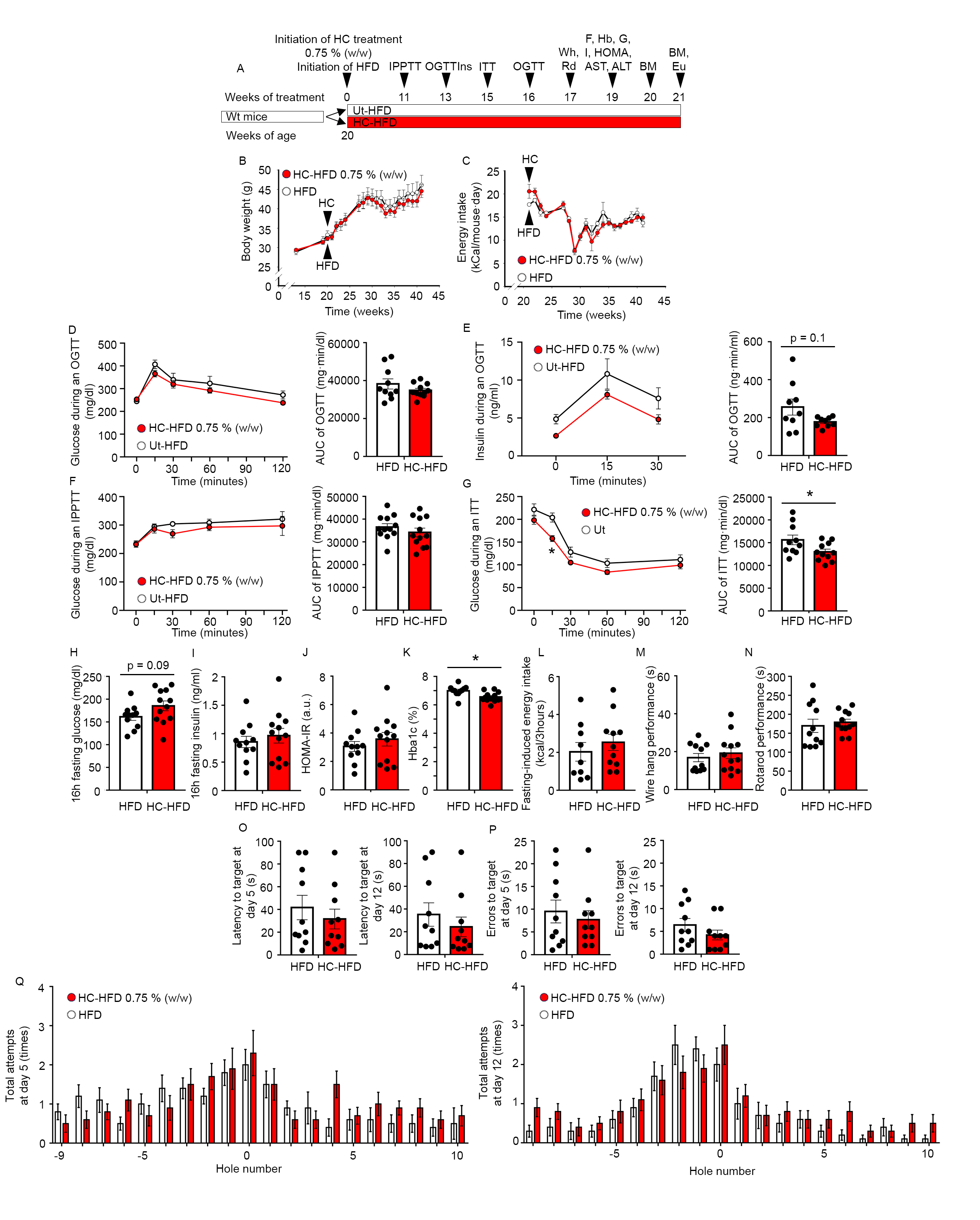

Supplement: Supplementary file 5 — Figure S5. [file ACEL-23-e14205-s007.jpg]

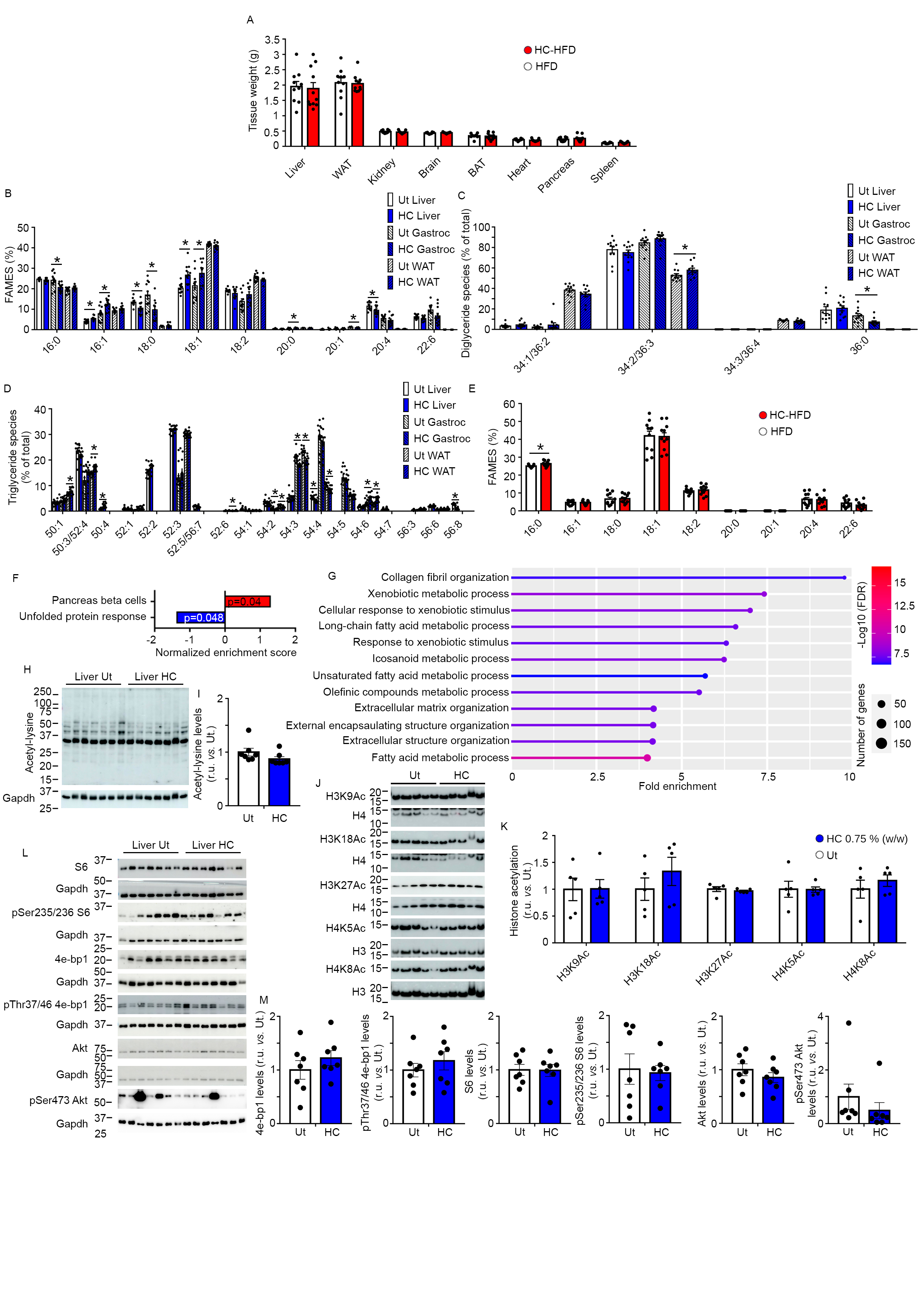

Supplement: Supplementary file 6 — Figure S6. [file ACEL-23-e14205-s008.jpg]

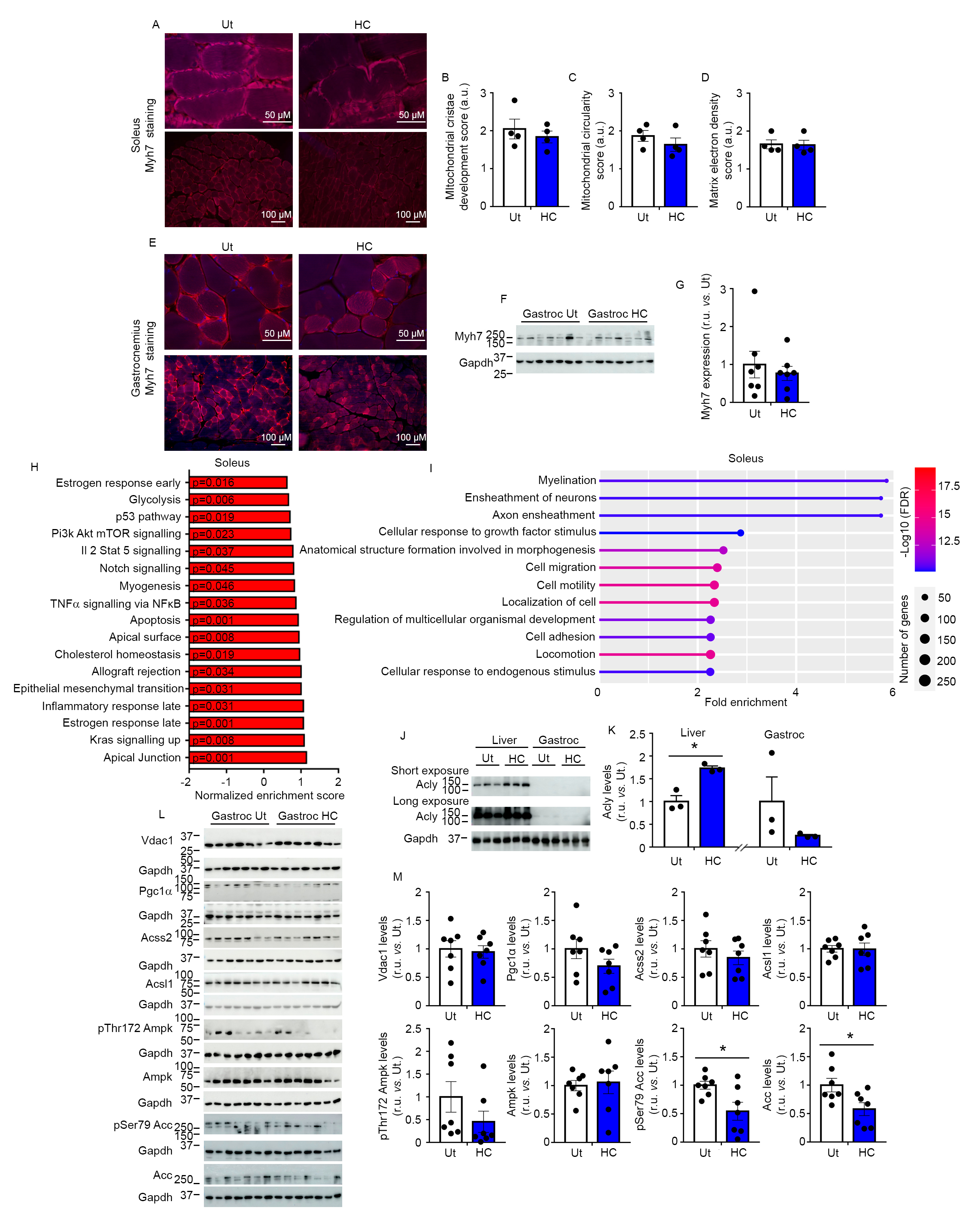

Supplement: Supplementary file 7 — Figure S7. [file ACEL-23-e14205-s003.jpg]

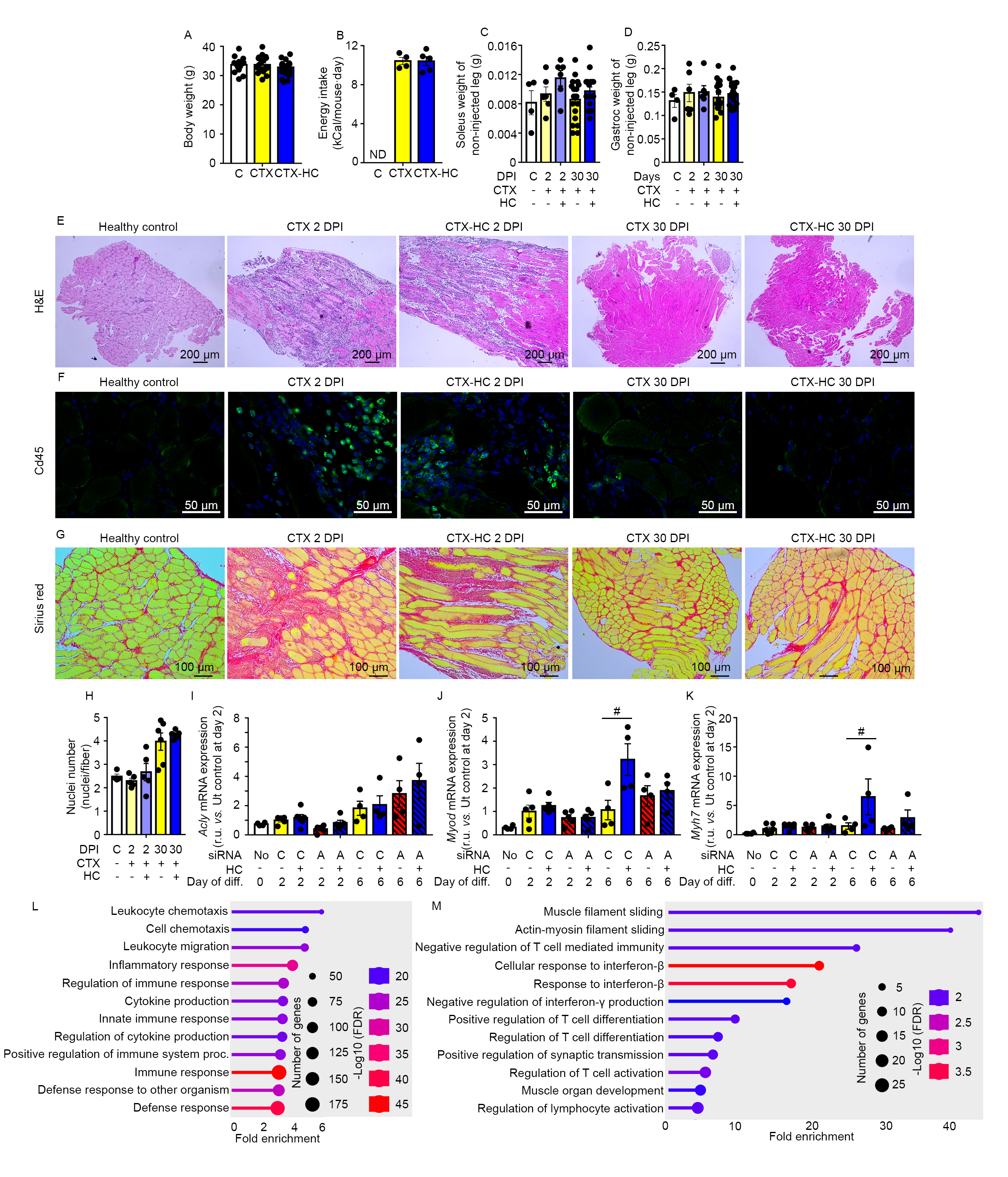

Supplement: Supplementary file 8 — Figure S8. [file ACEL-23-e14205-s001.jpg]
