# Supplementary material for: Hydroxycitrate delays early mortality in mice and promotes muscle regeneration while inducing a rich hepatic energetic status
Source: Aging Cell. 2024 May 17;23(9):e14205. doi: 10.1111/acel.14205 (PMC11488303; doi:10.1111/acel.14205)
Supplement: Supplementary file 9 — Appendix S1. [file ACEL-23-e14205-s009.docx]

**Supplementary figure legends.**

**Figure S1. Clinical parameters associated with phospho-active ACLY and ACLY expression in humans undergoing liver biopsies.** (A) Linear regression of hepatic pSer455 ACLY and free T4. n = 10 males and 18 females. Pearson correlation. (B) Linear regression of hepatic pSer455 ACLY and ALT. n = 12 males and 18 females. Pearson correlation. (C) Linear regression of hepatic pSer455 ACLY and digital pathology (% collagen). n = 11 males and 13 females. Pearson correlation. (D) Linear regression of hepatic ACLY and ferritin. n = 11 males and 17 females. Pearson correlation. (E) Linear regression of ACLY and Deugnier iron score. n = 11 males and 15 females. Pearson correlation. (F) Linear regression of hepatic ACLY and percentage of lymphocytes. n = 11 males and 17 females. Pearson correlation. (G) ACLY expression levels in female hypothyroid and non-hypothyroid subjects. n = 9 non-hypothyroid. n = 8 hypothyroid. Student´s t-test. Of note, none of the male subjects in the study was hypothyroid. (H) Linear regression of hepatic ACLY and hepatic pSer455 ACLY. n = 11 males and 16 females. Pearson correlation. r. u.: relative units. a.u.: arbitrary units. Data shown are individual biological values.

**Figure S2. Effects of ACLY inhibitors in AML12 hepatocytes.** AML12 cells were siRNA interfered for Acly expression and were treated with HC 1 mM, SB 10 µM or Bemp 30 µM for 16 hours. (A) Acly mRNA expression. C: siRNA control. A: siRNA Acly. n = 6. Kruskal Wallis plus Dunn´s post hoc test. (B) Representative western blots of Acly. n = 4 for siRNA control Ut, siRNA control HC, siRNA Acly Ut, n = 3 for siRNA control SB, siRNA control Bemp, siRNA Acly HC, siRNA Acly SB, siRNA Acly Bemp. (C-L) Volcano plots depicting the fold change and the statistical significance of transcripts. Over-expressed transcripts are highlighted in red and down-regulated transcripts are highlighted in blue. Reference line indicates the threshold of significance. n = 3. (M) Top 5 enriched terms by Metascape analysis in siRNA ACLY *vs.* siRNA control. n = 3. (N) Top 12 enriched processes by ShinyGO analysis in siRNA ACLY *vs.* siRNA control. n = 3. (O) Diagram exhibiting significantly modulated genes altered by Acly inhibitors in siRNA control AML12 cells. Upregulation (red), downregulation (blue), reciprocal regulation (black). n = 3. (P) Diagram exhibiting significantly modulated genes altered by Acly inhibitors in siRNA Acly AML12 cells. Upregulation (red), downregulation (blue), reciprocal regulation (black). n = 3. (Q) Diagram exhibiting significantly modulated genes altered by HC in siRNA Acly and siRNA control AML12 cells. Upregulation (red), downregulation (blue). n = 3. (R) Diagram exhibiting significantly modulated genes altered by SB in siRNA Acly and siRNA control AML12 cells. Upregulation (red), downregulation (blue). n = 3. (S) Diagram exhibiting significantly modulated genes altered by Bemp in siRNA Acly and siRNA control AML12 cells. Upregulation (red), downregulation (blue). n = 3. (T) 12 top significantly modulated canonical pathways by Ingenuity Pathway Analysis in siRNA Acly HC *vs.* siRNA control HC AML12 cells. Bars with positive z-scores are filled in red and bars with negative z-scores are filled with blue. n = 3. (U) 12 top significantly modulated canonical pathways by Ingenuity Pathway Analysis in siRNA Acly SB *vs.* siRNA control SB AML12 cells. Bars with positive z-scores are filled in red and bars with negative z-scores are filled with blue. n = 3. (V) 12 top significantly modulated canonical pathways by Ingenuity Pathway Analysis in siRNA Acly Bemp *vs.* siRNA control Bemp AML12 cells. Bars with positive z-scores are filled in red and bars with negative z-scores are filled with blue. n = 3. Ut: Untreated. HC: Hydroxycitrate. SB: SB-204990. Bemp: Bempedoic acid. C: control. Und: undetermined. Data shown are the means ± SEM. * p < 0.05 specific experimental condition *vs.* siRNA control.

**Figure S3. Effects of HC in energy intake in mice.** (A) Energy intake during the longevity assay in mice. n = 4 cages. Student´s t-test. HC: Hydroxycitrate. Ut: Untreated. Data shown are the means ± SEM. * p < 0.05 HC *vs.* Ut.

**Figure S4. HC does not alter neurocognition, exerts minor effects in glucoregulation and alters locomotor function in healthy diet.** (A) Graphical representation of the experimental model. BM: Barnes maze. F: Fasting-induced energy intake. Hb: Glycated hemoglobin. G: Glucose. I: Insulin. NOR: Novel object recognition. Rd: Rotarod. Wh: Wire hang. FC: Fear conditioning. IC: Indirect calorimetry. (B-G) Wild-type mice treated or not with HC were subjected to indirect calorimetry evaluation at week 71 of treatment. (B) Monitorization of spontaneous activity and daily spontaneous activity. n = 7. Repeated measures two-way ANOVA Bonferroni post hoc test for monitorization and Student´s t-test for daily determination. (C) Water intake during indirect calorimetry. n = 7 Ut, n = 4 HC. Student´s t-test. (D) Monitorization of energy expenditure and daily energy expenditure. n = 7. Repeated measures two-way ANOVA Bonferroni post hoc test for monitorization and Student´s t-test for daily determination. (E) Daily energy intake during indirect calorimetry. n = 7 Ut, n = 6 HC. Student´s t-test. (F) Monitorization of rearing activity and daily rearing activity. n = 7 Ut, n = 6 HC. Repeated measures two-way ANOVA Bonferroni post hoc test for monitorization and Student´s t-test for daily determination. (G) Monitorization of respiratory quotient and daily rearing quotient. n = 7. Repeated measures two-way ANOVA Bonferroni post hoc test for monitorization and Student´s t-test for daily determination. (H) Glucose during an OGTT and Area under the curve (AUC) of glucose levels during an OGTT at 21 weeks of treatment. n = 9. Repeated measures two-way ANOVA Bonferroni for glucose levels and Student´s t-test for AUC. (I) Insulin during an OGTT and AUC of insulin levels during OGTT at 24 weeks of treatment. n = 8 Ut, n = 10 HC. Repeated measures two-way ANOVA Bonferroni for glucose levels and Student´s t-test for AUC. (J) Glucose during an IPPTT and AUC of glucose levels during an IPPTT at 20 weeks of treatment. n = 10. Repeated measures two-way ANOVA Bonferroni for glucose levels and Student´s t-test for AUC. (K) Glucose during an ITT and AUC of glucose levels during an ITT at 22 weeks of treatment. n = 10. Repeated measures two-way ANOVA Bonferroni for glucose levels and Student´s t-test for AUC. (L) Glucose levels at 16 hours of fasting at 24 weeks of treatment. n = 10. Student´s t-test. (M) Insulin levels at 16 hours of fasting at 24 weeks of treatment. n = 10. Student´s t-test. (N) HOMA-IR index at 16 hours of fasting at 24 weeks of treatment. n = 10. Student´s t-test. (O) Serum ALT levels in fasting conditions at week 19 of treatment. n = 4 Ut, n = 4 HC, n = 4 HFD, n = 5 HC-HFD. Two-way ANOVA Tukey post hoc test. (P) Serum AST levels in fasting conditions at week 19 of treatment. n = 3 Ut, n = 4 HC, n = 4 HFD, n = 5 HC-HFD. Two-way ANOVA Tukey post hoc test. (Q) Percentage of glycated hemoglobin at 24 weeks of treatment. n = 10. Student´s t-test. (R) Fasting-induced energy intake at 23 weeks of treatment. n = 10. Student´s t-test. (S-U) Spatial memory was assessed by Barnes Maze at week 21-22 of treatment in mice fed with a healthy STD. (S) Latency to target at day 5 and at day 12. n = 10. Student´s t-test. (T) Errors to target at day 5 and at day 12. n = 10. Student´s t-test. (U) Total attempts to target at day 5 and at day 12. n = 10. Student´s t-test. (V) Fear conditioning performance during the training test (before any tone-shock pairing) and during the contextual test at week 29 of treatment. (W) Fear conditioning performance during the pre-tone period and the tone presentation in the cued test at week 29 of treatment. n = 10. Note, performance of one mouse on training day was not recorded due to technical reasons. Student´s t-test. (X) Discrimination index in novel object recognition at week 25 of treatment. n = 5. Student´s t-test. (Y) Wire hang performance at week 27 of treatment. n = 24 Ut, n = 20 HC. Student´s t-test. (Z) Rotarod performance at week 26 of treatment. n = 23 Ut, n = 20 HC. Student´s t-test. HC: Hydroxycitrate. Ut: Untreated. a.u.: arbitrary units. Data shown are the means ± SEM.* p < 0.05 HC *vs.* Ut. IU: international units. NOR: novel object recognition. Data shown are the means ± SEM. Unless otherwise highlighted * p < 0.05 HC *vs.* Ut.

**Figure S5. HC exerts mild positive effects in glucoregulation, and does not alter neurocognition and locomotor function in HFD.** (A) Graphical representation of the experimental model. F: Fasting-induced energy intake. Hb: Glycated hemoglobin. G: Glucose. I: Insulin. Rd: Rotarod. Wh: Wire hang. BM: Barnes maze. Eu: Euthanization. (B) Body weight during the course of the study performed in HFD. n = 11-12 on HFD. n = 12 HFD-HC. Student´s t-test. (C) Energy intake during the course of the study performed in HFD. n = 3 cages. Student´s t-test. (D) Glucose during an OGTT and AUC of glucose levels during an OGTT at week 16 of treatment. n = 10 HFD, n = 11 HC-HFD. Repeated measures two-way ANOVA Bonferroni for glucose levels and Student´s t-test for AUC. (E) Insulin during an OGTT and AUC of insulin levels during an OGTT at week 13 of treatment. n = 9. Repeated measures two-way ANOVA Bonferroni for glucose levels and Student´s t-test for AUC. (F) Glucose during an IPPTT and AUC of glucose levels during an IPPTT at week 11 of treatment. n = 12. Repeated measures two-way ANOVA Bonferroni for glucose levels and Student´s t-test for AUC. (G) Glucose during an ITT and AUC of glucose levels during an ITT at week 15 of treatment. n = 10 HFD, n = 11 HC-HFD. Repeated measures two-way ANOVA Bonferroni for glucose levels and Student´s t-test for AUC. (H) Glucose levels at 16 hours of fasting at week 19 of treatment. n = 11 HFD, n = 12 HC-HFD. Student´s t-test. (I) Insulin levels at 16 hours of fasting at week 19 of treatment. n = 11 HFD, n = 12 HC-HFD. Student´s t-test. (J) HOMA-IR index at 16 hours of fasting at week 19 of treatment. n = 11 HFD, n = 12 HC-HFD. Student´s t-test. (K) Percentage of glycated hemoglobin at week 19 of treatment. n =11 HFD, n = 12 HC-HFD. (L) Fasting-induced energy intake at week 19 of treatment. n = 9 HFD, n = 11 HC-HFD. Student´s t-test. (M) Wire hang performance on HFD at week 17 of treatment. n =11. Student´s t-test. (N) Rotarod performance on HFD at week 17 of treatment. n =11 HFD, n = 12 HC-HFD. Student´s t-test. (O-Q) Spatial memory was assessed by Barnes Maze at week 20-21 of treatment in mice fed with a HFD. (O) Latency to target at day 5 and at day 12. n = 10. Student´s t-test. (P) Errors to target at day 5 and at day 12. n = 10. Student´s t-test. (Q) Total attempts to target at day 5 and at day 12. n = 10. Student´s t-test. HC: Hydroxycitrate. Ut: Untreated. HFD: High fat diet. a.u.: arbitrary units. Data shown are the means ± SEM. Data shown are the means ± SEM.* p < 0.05 HFD *vs.* HC-HFD.

**Figure S6. Effects of HC in the liver of mice fed with a healthy STD and mice fed with an unhealthy HFD.** (A) Tissue weight at euthanization in mice fed with HFD at 41 weeks of age (21 weeks of treatment). n = 10 HFD, n = 11 HC-HFD. Student´s t-test. (B) Analysis of FAMES in liver, gastrocnemius and WAT. n = 10 for liver Ut, n =11 for liver HC, gastrocnemius and WAT. Student´s t-test. (C) Analysis of diglyceride species in liver, gastrocnemius and WAT. n = 11. Student´s t-test. (D) Analysis of triglyceride species in liver, gastrocnemius and WAT. n = 10 for liver and gastroc, n =11 for WAT. Student´s t-test. (E) Analysis of FAMES in liver of mice fed with a HFD. n = 11. Student´s t-test. (F) Enriched gene sets by GSEA analysis liver tissue. n = 5. (G) Top 12 enriched processes by ShinyGO analysis in liver tissue. n = 5. (H) Representative western blots of global hepatic acetyl-lysine modified proteins of mice treated with HC. n = 7. (I) Densitometric quantification of western blots shown in panel H. n = 7. Student´s t-test. (J) Representative western blots of hepatic acetyl-lysine modified histones of mice treated with HC. n = 5. (K) Densitometric quantification of western blots shown in panel J. n = 5. Student´s t-test. (L) Representative western blots of hepatic proteins of mice treated with HC. n = 7. (M) Densitometric quantification of western blots shown in panel L. n = 7. Student´s t-test. HC: Hydroxycitrate. Ut: Untreated. HFD: High fat diet. r. u.: relative units. Data shown are the means ± SEM. * p < 0.05 HC *vs.* Ut or HFD *vs.* HC-HFD.

**Figure S7. Effects on HC in the skeletal muscle of mice fed with a healthy STD.** (A) Immunohistochemical analysis of Myh7 expression in soleus tissue. n = 5. (B) Mitochondrial cristae development score. n = 4. Student´s t-test. (C) Mitochondrial circularity score. n = 4. Student´s t-test. (D) Mitochondrial matrix electron density score. n = 4. Student´s t-test. (E) Immunohistochemical analysis of Myh7 expression in gastrocnemius. n = 5. (F) Representative western blots of Myh7 of mice treated with HC in the gastrocnemius. n = 7. (G) Densitometric quantification of western blots shown in panel F. n = 7. Student´s t-test. (H) Enriched gene sets by GSEA analysis the soleus. n = 5. (I) Top 12 enriched processes by ShinyGO analysis in soleus. n = 5. (J) Evaluation of Acly levels by western blot in the liver and the gastrocnemius of mice treated with HC. n = 3. (K) Densitometric quantification of western blots shown in panel J. Acly expression in each tissue was relativized to the untreated control. n = 3. Student´s t-test. (L) Representative western blots of gastrocnemius mice treated with HC. n = 7. (M) Densitometric quantification of western blots shown in panel K. n = 7. Student´s t-test. HC: Hydroxycitrate. Ut: Untreated. r. u.: relative units. Data shown are the means ± SEM. * p < 0.05 HC *vs.* Ut.

**Figure S8. Effects of HC in mice injected with CTX**. (A) Body weight of mice injected with CTX at 28 DPI. n = 16 C, n = 19 CTX, n = 20 CTX-HC. One-way ANOVA plus Tukey post hoc test. (B) Energy intake of mice injected with CTX. n = 4 cages CTX, n = 5 cages CTX-HC. Student´s t-test. (C) Soleus weight of non-injected leg. n = 4 C, n = 6 CTX 2 DPI, n = 6 CTX-HC 2 DPI. n = 19 CTX 30 DPI, n = 17 CTX-HC 30 DPI. One-way ANOVA plus Tukey post hoc test. (D) Gastrocnemius weight of non-injected leg. n = 4 C, n = 6 CTX 2 DPI, n = 6 CTX-HC 2 DPI, n = 19 CTX 30 DPI, n = 19 CTX-HC 30 DPI. One-way ANOVA plus Tukey post hoc test. (E) Representative images of soleus hematoxylin and eosin staining. n = 4 healthy control, n = 5 CTX 2 DPI, n = 5 CTX-HC 2 DPI, n = 6 CTX 30 DPI, n = 6 CTX-HC 30 DPI. (F) Representative images of soleus Cd45^+^ immune infiltration staining. n = 4 C, n = 5 CTX 2 DPI, n = 5 CTX-HC 2 DPI, n = 6 CTX 30 DPI, n = 6 CTX-HC 30 DPI. (G) Representative images of soleus Sirius red staining. n = 4 healthy control, n = 6 CTX 2 DPI, n = 5 CTX-HC 2 DPI, n = 6 CTX 30 DPI, n = 6 CTX-HC 30 DPI. (H) Nuclei per fiber in soleus. n = 4 C, n = 5 CTX 2 DPI, n = 5 CTX-HC 2 DPI, n = 6 CTX 30 DPI, n = 5 CTX-HC 30 DPI. One-way ANOVA plus Tukey post hoc test. (I-K) C2C12 cells were exposed to siRNA Acly, treated with 1 mM HC and differentiated into myoblasts. mRNA expression was determined at different time points. (I) Acly expression. n = 4 non-differentiated, n = 6 siRNA control at 2 days of differentiation (Day of diff., or DOD), n = 6 siRNA control at 2 DOD treated with HC, n = 5 siRNA Acly at 2 DOD n = 5 siRNA Acly at 2 DOD treated with HC, n = 4 siRNA control at 6 DOD, n = 4 siRNA control at 6 DOD treated with HC, n = 4 siRNA Acly at 6 DOD, n = 4 siRNA Acly at 6 DOD treated with HC. (J) Myod expression. n = 4 non-differentiated, n = 5 siRNA control at 2 DOD, n = 5 siRNA control at 2 DOD treated with HC, n = 5 siRNA Acly at 2 DOD, n = 5 siRNA Acly at 2 DOD treated with HC, n = 4 siRNA control at 6 DOD, n = 4 siRNA control at 6 DOD treated with HC, n = 4 siRNA Acly at 6 DOD, n = 4 siRNA Acly at 6 DOD treated with HC. (K) Myh7 expression. n = 4 non-differentiated, n = 6 siRNA control at 2 DOD, n = 6 siRNA control at 2 DOD treated with HC, n = 5 siRNA Acly at 2 DOD, n = 6 siRNA Acly at 2 DOD treated with HC, n = 4 siRNA control at 6 DOD, n = 4 siRNA control at 6 DOD treated with HC, n = 4 siRNA Acly at 6 DOD, n = 4 siRNA Acly at 6 DOD treated with HC. (L-M) Top 12 enriched processes by ShinyGO analysis in at 2 DPI and 30 DPI. (L) CTX HC 2 DPI *vs.* CTX 2 DPI. n = 3. (M) CTX HC 30 DPI *vs.* CTX 30 DPI. n = 3. Gastroc: Gastrocnemius. nd: not determined. HC: Hydroxycitrate. Ut: untreated. C: healthy control mice not injected with CTX or siRNA control. A: siRNA Acly. CTX: cardiotoxin. DPI: days post injury. r. u.: relative units. Data shown are the means ± SEM. * p < 0.05 CTX *vs.* C. # p < 0.05 CTX-HC *vs.* CTX or HC-C *vs.* C.

Table S1. List of resources for materials used in this work.

| Antibodies | | | |
| --- | --- | --- | --- |
| Antibody | SOURCE | IDENTIFIER | DILUTION |
| Anti-Rabbit IgG HRP-linked | Cell Signaling Technology | 7074 | 1:5000 |
| Alexa fluor 594 goat anti-mouse | Thermo Fisher | A32742 | 1:500 |
| Alexa fluor 488 goat anti-rabbit | Thermo Fisher | A11034 | 1:500 |
| Anti-Mouse IgG BP-HRP | Santa Cruz Biotechnology | Sc-516102 | 1:2000 |
| Gapdh | Cell Signaling Technology | Cat# 2118, RRID:AB_561053 | 1:5000 |
| pSer455 Acly | Cell Signaling Technology | 4331 | 1:1000 |
| pSer455 Acly | Sigma-Aldrich | SAB4504020 | 1:100 |
| Acly | Cell Signaling Technology | 4332 | 1:1000 |
| Acly | ABCAM | Ab40793 | 1:250 |
| Acss2 | Cell Signaling Technology | 3658 | 1:1000 |
| pThr172 Ampk | Cell Signaling Technology | Cat# 2535, RRID:AB_331250 | 1:1000 |
| Ampk | Cell Signaling Technology | Cat# 2532, RRID:AB_330331 | 1:1000 |
| pSer 235/236 S6 | Cell Signaling Technology | 4856 | 1:1000 |
| S6 | Cell Signaling Technology | 2217 | 1:1000 |
| Myh7 | Santa Cruz Biotechnology | Sc-53090 | 1:200 |
| Fas | Santa Cruz Biotechnology | Sc-55580 | 1:1000 |
| 4E-bp1 | Cell Signaling Technology | 9452 | 1:1000 |
| pThr37/46 4E-bp1 | Cell Signaling Technology | 2855 | 1:1000 |
| Acc1 | Cell Signaling Technology | 3676 | 1:1000 |
| pSer79 Acc1 | Cell Signaling Technology | 11818 | 1:1000 |
| H3K9 Ac | Cell Signaling Technology | 9649 | 1:1000 |
| H4 | Cell Signaling Technology | 13919 | 1:1000 |
| H3K18 Ac | Cell Signaling Technology | 9675 | 1:1000 |
| H3K27 Ac | Cell Signaling Technology | 8173 | 1:1000 |
| H4K5 Ac | Cell Signaling Technology | 8647 | 1:1000 |
| H3 | Cell Signaling Technology | 4499 | 1:1000 |
| H4K8 Ac | Cell Signaling Technology | 2594 | 1:1000 |
| Pan Acetyl lysine | Cell Signaling Technology | 9441 | 1:1000 |
| Akt | Cell Signaling Technology | 9272 | 1:1000 |
| pSer473 Akt | Cell Signaling Technology | 9271 | 1:1000 |
| Anti-Rabbit IgG (whole molecule)–Peroxidase | Sigma-Aldrich | A9169 | 1:5000 |
| Vdac1 | Cell Signaling Technology | Ab15895 | 1:1000 |
| Pgc1α | Santa Cruz Biotechnology | Sc-517380 | 1:200 |
| Acsl1 | Cell Signaling Technology | 9189 | 1:1000 |
| Cd45 | ABCAM | Ab10558 | 1:200 |
| Chemicals, Peptides, and Recombinant Proteins | | |  |
| Tripotassium hydroxycitrate | Sigma-Aldrich | 59847 |  |
| Tripotassium hydroxycitrate | Hangzhou Dayangchem Co. LTD | Not available |  |
| SB-204990 | Tocris and Sreeni Labs | 4962/10 |  |
| Bempedoic acid/ETC-1002 | Cayman chemicals | 26409 |  |
| Cardiotoxin | Latoxan | L8102 |  |
| siRNA Acly ON-TARGETplus SMART pool | Cultek | Not available |  |
| siRNA Acly ON-TARGETplus non-targeting pool | Cultek | Not available |  |
| Critical Commercial Assays | | |  |
| Hemoglobin A1c (HbA1c) Assay kit | Crystal Chem | Cat# 80099 |  |
| EnzyChrom Triglyceride Assay Kit | BioAssay Systems | Cat# ETGA-200 |  |
| Insulin kit | Crystal Chem | 90080 |  |
| MTT kit | Sigma-Aldrich | 11465007001 |  |
| Deposited Data | | |  |
| RNAseq data | Gene Expression Omnibus | GSE232837 |  |
| Experimental Models: Cell Lines | | |  |
| AML12 cells | ATCC | CRL-2254 |  |
| C2C12 cells | ATCC | CRL-1772 |  |
| Experimental Models: Organisms/Strains | | |  |
| Wild type mice | Charles River | C57BL6 |  |
| Oligonucleotides | | |  |
| Ms Acly Fw | 5´TTCGTCAAACAGCACTTCC 3´ | Not available |  |
| Ms Acly Rw | 5´ATTTGGCTTCTTGGAGGTG 3´ | Not available |  |
| Ms Rps29 Fw | 5’-GGAGTCACCCACGGAAGTT-3’ | Not available |  |
| Ms Rps29 Rw | 5’-CATGTTCAGCCCGTATTTGC-3’ | Not available |  |
| Ms Myod Fw | 5´-GAGCGCATCTCCACAGACAG-3´ | Not available |  |
| Ms Myod Rw | 5´-AAATCGCATTGGGGTTTGAG-3´ | Not available |  |
| Ms Myh7 Fw | 5´-GTTTCCTTACTTGCTACCCTCAG-3´ | Not available |  |
| Ms Myh7 Rw | 5´-TGGATTCTCAAACGTGTCTAGTG-3´ | Not available |  |
| Software and Algorithms | | |  |
| ImageJ | NIH | RRID:SCR_001935 |  |
| SigmaPlot 14.5 | SigmaPlot | RRID:SCR_003210 |  |
| Graph Pad Prism 7 | Graphpad | RRID:SCR_002798 |  |
