# Supplementary material for: Hydroxycitrate delays early mortality in mice and promotes muscle regeneration while inducing a rich hepatic energetic status
Source: Aging Cell. 2024 May 17;23(9):e14205. doi: 10.1111/acel.14205 (PMC11488303; doi:10.1111/acel.14205)
Supplement: Supplementary file 10 — Appendix S2. [file ACEL-23-e14205-s010.docx]

**Online Methods**

**Mice, diets and *in vivo* treatments**

Mice experimentations were approved by the CABIMER Animal Committee and performed in accordance with the Spanish law on animal use RD 53/2013 and the EU Directive (2010/63/EU) for animal research.

For the longevity assay 8-week old wild-type male C57BL/6 mice were purchased from Charles River. Mice were housed in individually ventilated cages (Tecniplast, Buguggiate, Italy). Souralit plus 29/12 bedding (Souralit, Gerona, Spain) was sterilized by autoclave and added to each cage. Mice were group‐housed at 3-7 mice per cage. Mice were maintained on a 12 hours light/dark cycle and had *ad libitum* access to rodent chow TD2914 (Envigo, Barcelona, Spain) and water until the initiation of the treatments. In animals treated or not with HC, mice were distributed in different groups at 20-weeks of age and treatments were initiated at that moment: (1) standard TD2914 diet (STD) with the following percentages of calories distribution: 67 % from carbohydrates, 20 % from proteins and 13 % from fats. (2) STD + 7.5g/Kg of HC (STD-HC). (3) A cholesterol-free high-fat diet (HFD) TD.06414 (Envigo, Barcelona, Spain) with the following percentages of calories distribution: 21.4 % from carbohydrates, 18.3 % from proteins and 60.3 % from fats (37 % saturated, 47 % monounsaturated and 16 % polyunsaturated). (4) HFD + 7.5g/Kg of HC (HFD-HC). At week 41 of life, a cohort of mice was euthanized at 16 hour of fasting for the *ex vivo* analyses. Dead animals on the longevity assay were necropsied at time of death.

For the cardiotoxin model of muscle damage, 6±1 month-old wild-type male C57BL/6 mice were injected or not with 10 µM CTX (Latoxan) ([Vinel et al., 2018](#_ENREF_40)). The day of the injection, mice were anesthetized with isoflurane inhalation. Then, CTX was injected in the belly of the left hind limb to reach the gastrocnemius and the soleus (100 µl total volume) using a 31-gauje needle. Subsequently, mice were supplemented or not with HC at 7.5g/Kg of HC in STD. Mice were subjected to an evaluation of physical health using wire hang (day 10), treadmill (day 16), rotarod (21), and grip strength (day 27). On day 28 post-CTX injection the control group (not previously exposed to CTX) was divided into three groups to evaluate early (2 days) responses to CTX injection. These mice were anesthetized with isoflurane inhalation and received or not an injection of 10 µM CTX in the belly of the left hind limb to reach the gastrocnemius and the soleus (100 µl total volume) using a 31-gauje needle. Subsequently, these mice were supplemented or not with HC at 7.5g/Kg of HC in STD for 2 days. Mice were euthanized at 2 days or 30 days post CTX injection, muscles were snap frozen in liquid nitrogen or embedded in optimal cutting temperature compound in isopentane cooled with liquid nitrogen.

**Liver biopsies and immunohistochemistry of human samples**

Samples were obtained from the Clinic University Hospital of Valencia following approval from the institutional review board. The cohort consisted in 30 liver biopsies used for the clinical diagnosis of the subjects. Sex was assigned based on the physical characteristic of the patients and their medical record. Samples were evaluated by histopathology and clinical parameters according to the protocols of the Clinic University Hospital of Valencia ([Marti-Aguado et al., 2021](#_ENREF_23)). 4 µm sections from formalin-fixed paraffin embedded samples were prepared for immunohistochemical staining. Antigen retrieval was performed with citrate buffer pH 6 (Sigma-Aldrich) by boiling the slides for 20 minutes in an autoclave followed by cooling at room temperature for 10 minutes. The sections were then incubated with blocking solution consisting in PBS with Triton-X (0.1 % v/v), BSA 0.3 % and normal goat serum 10 % at room temperature for 1 hour. Incubations with primary antibodies were performed at the following dilutions: Anti-ATP citrate lyase antibody [EP704Y] (ab40793, Abcam) at 1:250, Anti-phospho-ATP-Citrate Lyase (pSer454) (SAB4504020, Sigma-Aldrich) at 1:100, 4 ºC overnight in a humidified chamber. The slides were then washed in PBS 1x, Tx 0.1 %, BSA 0.3 % and incubated with secondary antibody according to VECTASTAIN® ABC-HRP Kit, Peroxidase (Rabbit IgG) (Vector Labs). The staining was developed with DAB Substrate Kit, Peroxidase (HRP), with Nickel (Vector Labs). The evaluation of integrated density was performed using ImageJ.

**Indirect calorimetry**

Indirect calorimetry was assayed in a OxyletPro system (PanLab Harvard Apparatus). Mice were in individual cages with water and food available *ad libitum* and maintained at ~22 °C under a 12:12 hours light:dark cycle (light period 08:00-20:00 hours). Oxygen and carbon dioxide concentrations were determined at the inlet and outlet of the sealed cages. Each chamber was measured for 60 seconds at 10 minutes intervals and data were recorded for ~48 hours total. Locomotor activity was monitored using infrared photocell beams for rearing, and a sensor platform for activity. Food and water intake was automatically monitored using specific sensors in individual cages.

**Glucoregulation tests**

For OGTTs, mice were fasted for 6 hours at 10 a.m. and received an oral gavage of glucose (3 g/kg). For IPPTT, mice were fasted for 6 hours from 10 a.m. and received an intraperitoneal injection of sodium pyruvate (2 g/kg). For the ITT, mice were fasted for 3 hours from 10 a.m. and received an intraperitoneal injection of insulin (1.5 IU/kg). For the HOMA-IR, mice were fasted from 6 p.m. and determinations were performed at 16 hours of fasting. To determine glucose levels, blood samples were taken by venipuncture using a Precision Xceed glucometer (Abbott, Madrid, Spain). Insulin was measured in plasma using ELISA kits (Crystal Chem, Downers Grove, IL, USA).

**Fasting-induced energy intake**

Mice were fasted for 16 hours. Subsequently, food was provided *ad libitum* and food intake was determined at 3 hours.

**Glycated hemoglobin**

HbA1c levels were determined in blood samples according to the indications of the manufacturer (Crystal Chem).

**ALT and AST determination**

Serum levels of AST and ALT were analyzed using a Cobas Integra 400 plus automated analyzer (Roche Diagnostics).

**Barnes maze**

On the pre-training trial, mice were pre-trained to enter the escape box, guiding them to the escape box. Mice remained in the box for 2 minutes. Then, training trials were initiated (4 days). Mice were trained three trials per day and trials were separated by 15 minutes. Mice were allowed to explore the maze for up to 3 minutes and were guided to the escape box. The buzzed was turned off when mice entered the box and mice remained in the box for 1 minute. The following day, subjects received a probe trial for 90 seconds to determine short-retention memory. During the probe trial the escape box was removed. Primary latency and total attempts were recorded. Without further training mice were tested for another probe trial for 90 seconds 7 days after to determine long-term retention memory.

**Fear conditioning**

Neurofunction was evaluated by the fear conditioning paradigms in a three-days protocol, using a StartFear Combined system (Panlab, Harvard Apparatus, Barcelona, Spain). The first day (training), animals were placed in the test chamber and allowed to explore for 3 minutes. Mice were then exposed to a 30 seconds tone (80 dB) followed by a 2 seconds foot shock (0.2 mA). Animals received 3 additional tone-shock pairings, with a 70 seconds interval between pairing. The second day (contextual testing), mice were placed in the same test chamber and allowed to explore for 3 minutes in the absence of tone and foot shock. Freezing was measured across the 3 minutes period. The third day (cued testing), mice were placed in the same test chamber, but with an altered environment by changing the chamber walls and ground, and mice were allowed to explore the chamber for 3 minutes. Then, the auditory cue (80 dB tone) was presented for 3 minutes. Freezing was measured before and during the presentation of the tone. Data were analyzed using the Packwin v2.0 software (Panlab).

**Novel object recognition**

Novel object recognition test was performed with a long habituation phase, using odorless objects that do not retain any olfactory cues ([Leger et al., 2013](#_ENREF_19)). Briefly, mice were placed into the testing chamber for three consecutive days for habituation. The following day, mice were placed into the same chamber containing two identical objects (Object A) for 10 minutes, or until the object exploration time in both objects reached 20 seconds. After 24 hours, mice were placed back into the chamber containing the familiar object (Object A) and a novel object (Object B). Mice were allowed to explore the objects for 10 minutes, or until the object exploration time reached 20 seconds in both objects. Total exploration time of familiar and novel objects were recorded and the discrimination index was calculated.

**Physical performance**

Mice were evaluated for physical performance using different protocols. For wire hang test, mice were allowed to grip a horizontal 1 mm wire with their four paws up to 300 seconds, and the latency to fall was determined. Results shown are the averages of three trials per mouse. For rotarod tests, results are presented as the time to fall from an accelerating rotarod (4–40 rotations per minute over 5 minutes) using a Ugo Basile equipment. Mice were given a 1-minute trial for habituation at 4 rotations per minute the day before the experiment. Results shown are the averages of three trials per mouse. For treadmill test, results are expressed as the distance run until exhaustion in a PanLab equipment. The treadmill was horizontal and mice were given a 5-minute trial for habituation at 4 m/minute the day before the experiment. The following day each mouse was given a single trial at 7 m/minute for 3 minutes, 12 m/minute for 4 minutes, 15 m/minute for 18 minutes, 19 m/minute for 25 minutes and 24 m/minute for 30 minutes. Grip strength was determined in a Ugo Basile equipment. Mice were placed in a grid until they gripped the grid with their four paws. Then mice were gently pulled by the tail until they could no longer hold the grid. Results shown are the average of three trials per mouse.

**Lipid analyses**

Lipids were extracted from ~20 mg of tissue using hexane-isopropanol as previously described ([Hara & Radin, 1978](#_ENREF_12)). In brief, lipids were transmethylated with acid methanol. After cooling, heptane was added and the samples were mixed. GC analyses were performed on a Hewlett–Packard 5890A gas chromatograph for healthy-fed mice (Palo Alto, CA, USA) or Agilent 7890 system for HFD-fed mice with a Supelco SP-2380 capillary column of fused silica (30 mm length, 0.25 mm i.d., 0.20 μm film thickness) (Bellefonte, PA, USA). Hydrogen was used as the carrier gas and the oven was set at 170 ºC for HFD-fed mice and 180 °C for healthy-fed mice. Fatty acids were identified using standards. For glycerolipid identifications, lipids were fractionated to separate neutrals from polar lipids applying a modification of the method of Nash and Frankle ([Nash & Frankel, 1986](#_ENREF_28)). Total lipids dissolved in 2 ml chloroform were loaded onto a 0.5 silica gel cartridge (Lichrolut, Merk, Darmstadt, Germany) equilibrated in the same solvent. Then, neutral lipids were eluted with 10 ml chloroform. The solvent was removed under nitrogen and samples were dissolved in the same volume of acetone/acetonitrile 60:40 and stored at -20 ºC. The glycerolipid analysis was carried out in a Waters e2695 separation module equipped with a 2420 evaporative light scattering (ELS) detector and an Acquity QDs mass spectrometry (MS) detector from Waters operating in parallel, so simultaneous ELS and MS records were obtained. For neutral lipids analysis a SunFire C18 Column, 100Å, 3.5 µm, 4.6 mm X 150 mm from Waters was used. The sample was eluted applying a binary gradient. The solvent A consisted in acetone/acetonitrile 1:1 containing 0.1 % formic acid; the solvent B was acetone containing 0.1 % formic acid. The column was equilibrated in 100 % solvent A, then the sample was injected and the proportion of solvent B was increased to 90 % in 15 minutes. The solvent was kept at 90 % solvent B for 5 minutes more. Then, the column was re-equilibrated in 100 % solvent for 5 minutes prior the next injection. The composition of neutral lipids was quantified using the ELS record, whereas the peaks were identified by comparing their retention times with those from commercial standards and according to their MS records, which provided the MW of the corresponding sodium adducts of the eluted diacylglycerols or triacylglycerols. The ELS detector operated using a nebulizer temperature of 25 °C and a tube temperature of 52 °C with a nitrogen pressure of 25 psi (1.72 Bar). The MS detector operated in positive scan within a mass range from 500 to 1000 Da. The cone voltage was 15V, whereas the capillary voltages were 1.5 kV (positive) and 0.8 kV (negative).

**Transmission electron microscopy**

Fresh soleus muscles were fixed in 2.5 % glutaraldehyde and 2 % paraformaldehyde in 0.1 M phosphate buffer for 72 hours at 4 ºC before processing for electron microscopy. To improve the post-fixing and embedding process of the samples, 200 µm sections were cut on a vibratome Leica VT-1000 (Leica, Heidelberg, Germany). Muscle samples were post-fixed with 2 % osmium, rinsed, dehydrated and embedded in Durcupan resin (Fluka, Sigma-Aldrich, St. Louis, USA). Ultrathin sections (60 – 80 nm) were cut with an Ultracut UC-6 (Leica microsystems, Wetzlar, Germany) with a diamond knife, stained with lead citrate (Reynold’s solution) and examined under a transmission electron microscope FEI Tecnai G2 Spirit BioTwin (ThermoFisher Scientific company, Oregon, USA). All images were acquired with a Xarosa digital camera (EMSIS GmbH, Münster, Germany) controlled by Radius software Version 2.1. Images were used to determine different morphological parameters of mitochondria, including circularity, matrix electron density and cristae developed. The criteria used for mitochondrial scores were based on previous reports ([Vue et al., 2023](#_ENREF_41)), with minor modifications to improve clarity. For mitochondrial circularity; Score 0: over 75% of mitochondria were circular, score 1: over 50% of the mitochondria were circular, score 2: over 25% of the mitochondria were circular, score 3: no circular mitochondria were visible. For matrix electron density; score 0: over 25% of mitochondria were electron dense, score 1: over 50% of mitochondria were electron dense, score 2: over 75% of mitochondria were electron dense. For cristae development; score 0: no sharply defined cristae were visible, score 1: over 75% of the mitochondrial area lacked cristae, score 2: over 50% of the mitochondrial area lacked cristae, score 3: over 25% of the mitochondrial area lacked cristae, score 4: less than 25% of the mitochondrial area lacked defined cristae.

**Histological and immunohistochemistry analyses in mouse tissues**

Dissected tissues were fixed in 4 % paraformaldehyde and embedded in paraffin. Liver, soleus and red gastrocnemius sections were cut at 6 µm thickness. Quantifications of sections were performed blinded to the treatment groups. Hematoxylin and eosin staining was performed to evaluate tissue architecture, immune infiltration and nuclear counting. Fusion index was defined as the average number of nuclei inside myotubes as a percentage of total number of nuclei. Multinucleated index was defined as the number of nuclei in multinucleated fiber cells as a percentage of total nuclei in fibers. PAS reagent was used to stain glycogen according to the standard protocols of the histology core facility of CABIMER. The analysis was performed on a range of 24-123 images per experimental group. PAS score was determined by assigning an equally weighted arbitrary score of 0 (no visible PAS staining) to 20 (maximal PAS staining) for each image and averaging the score of each experimental group. Sirius red staining was performed to determine collagen depositions, indicative of fibrosis. Images were taken using optical microscopy and polarized light microscopy to determine preferentially the interaction of the Sirius red dye with type I collagen (red fibers) and type III collagen (green fibers) ([Bonomini, Rodella, Moghadasian, Lonati, & Rezzani, 2013](#_ENREF_5); [Liu et al., 2021](#_ENREF_20)). Cellular CSA was determined using a range of 32-274 cells per individual for the soleus and a minimum of 300 cells per individual for the gastrocnemius. For Myh7 immunofluorescence analyses, tissues were washed and permeabilized with PBS with Triton X 100 (0.1 % v/v). After 1 hour blocking in PBS with Triton X 100 (0.1 % v/v) with 5 % normal goat serum at room temperature, sections were incubated overnight at 4°C with Myh7 antibody (sc-53090, Santa Cruz Biotechnology). Subsequently, slides were incubated with Alexa fluor 594 goat anti-mouse (A32742, Thermo Fisher) in PBS with Triton X 100 (0.01 % v/v) for 2 hours at room temperature followed by Hoechst-nuclear staining. Soleus sections were washed and permeabilized with citrate buffer with Tween-20 (0.05 % v/v). After 1 hour blocking in PBS with Triton X 100 (0.25 % v/v) with 3 % skim milk and 10 % normal goat serum at room temperature, sections were incubated overnight at 4°C with Cd45 antibody (Ab10558, Abcam). After that, tissues were incubated with Alexa fluor 488 goat anti-rabbit (A11034, Thermo Fisher) in PBS with Triton X 100 (0.25 % v/v) with 3 % milk and 10 % normal goat serum for 1 hour at room temperature, followed by mounting slides with Fluoroshield mounting medium with DAPI (Ab104139, Abcam). Positive stained area and cell area was evaluated using ImageJ. The area of individualized fibers was quantified for the analyses of cell size in the soleus. For larger tissues (*i.e.* gastrocnemius), total fiber area was divided by the number of fibers in the area.

**Liver triglycerides**

Triglyceride levels were determined using an EnzyChrom Triglyceride Assay Kit according to the instructions of the manufacturer (BioAssay Systems, ETGA-200).

**RNA-seq analyses**

RNA extraction was performed using RNAeasy mini Kit (Qiagen). RNA-seq libraries were made with the Illumina stranded mRNA prep ligation kit following the corresponding user guide. The libraries were sequenced on NovaSeq 6000 SP. Quality control Analyses, FASTQ generation files, adapters trimming and filtering of sequencing were performed using the manufacturer’s software BaseSpace and followed by FASTQ toolkit v1.0.0. FastQ files generated were aligned to mouse genome (library UCSC mm10) using RNAseq Alignment (Illumina) V.2.0.2, based on STAR Alignment. Then, Gene counts were used as normalized gene expression (TPM) and regularized log-transformed gene count for different analysis. Gene counts were analyzed using DEseq2 Illumina package (v1.1), and were normalized using DESeq2’s median of ratios for differential expressed genes. Gene Counts of differentially expressed genes with a p < 0.05 and Log2 of fold-change ≥ 1 or ≤ -1 of each comparative set were included in the heat map. Unit variance scaling was applied to rows (genes). Also, both rows (genes) and columns (samples) were clustered using correlation distance and average linkage. Gene counts were used in GSEA (v4.3.2) and ShinyGO (v0.76). DEseq results (Log2 of fold-change and p values) were used in IPA. For Metascape (v3.5) and ShinyGO (v0.77), the list of significantly modulated genes was used to analyze transcriptomic data.

**Western blot**

AML12 cells, livers and entire gastrocnemius were lysed in radioimmunoprecipitation assay buffer (50 mM Tris–HCl (pH 7.5), 150 mM NaCl, 1 % sodium deoxycholate, 0.1 % SDS, 1 % NP-40) with 10 μM trichostatin, 10 mM nicotinamide, 50 mM sodium butyrate, protease and phosphatase P0044, P5726 and P8340 (Sigma-Aldrich). Histone isolations were processed directly. Western blots were performed according to standard methods, which involved incubation with a primary antibody of interest, followed by incubation with a horseradish peroxidase-conjugated secondary antibody and enhanced chemiluminescence (Table S1). Blots were quantified with ImageJ, and the bands of interest were normalized to Gapdh staining.

**Acly activity**

Acly activity was determined as described with minor modifications ([MacDonald, Longacre, Warner, & Thonpho, 2013](#_ENREF_21)). In brief, liver tissue was lysed in ice-cold 220 mM mannitol, 70 mM sucrose, 5 mM potassium HEPES buffer, pH 7.5 containing 1 mM dithiothreitol. Lysates were centrifuged at 600 x *g* for 10 minutes to precipitate the nuclei and debris. Then, the supernatant was centrifuged at 5500 x *g* for 10 minutes to precipitate the mitochondrial fraction and the supernatant was then centrifuged 20000 x *g* for 20 minutes to generate a cytosolic fraction. Enzymatic activity was measured in 5 mM citrate, 0.3 mM coenzyme A, 3 mM ATP, 0.15 mM NADH, 10 mM MgCl_2_, 10 mM dithiothreitol, and 6 units/ml of malate dehydrogenase in 100 mM Tris chloride buffer, pH 8.5 at 37º C in the presence or not of 5 mM HC. NADH absorbance was monitored at 340 nm during 1 minute for background, after which 5 mM citrate was added to determine Acly activity.

**AML12 culture, C2C12 culture and siRNA interference**

AML12 cells were obtained from ATCC (CRL-2254) and were maintained and propagated in DMEM/nutrient mixture F-12 Ham (D8437 Sigma-Aldrich) with 10 % fetal bovine serum, pen-strep, ITS Liquid Media Supplement, and 0.1 μM dexamethasone. At 4 hours post-seeding cells were treated with siRNA Acly or control siRNA (Cultek). At 24 hours post siRNA interference, cells were treated with HC 1 mM, SB 10 µM or Bemp 30 µM for 16 hours. Then samples were snap frozen and maintained at -80 ºC or processed for various experiments. Evaluation of lipid content was performed using Oil red O staining. Quantification of lipid content was performed after isopropanol solubilization at 510 nm. MTT activity was determined adding the reagent to the cells. Quantification of metabolic activity was performed after SDS (10 %) solubilization by optical density at 575 nm with a reference wavelength of 690 nm using a Varioskan Flash spectrophotometer (Thermo Scientific, Spain).

C2C12 cells were obtained from ATCC (CRL-1772) and were maintained and propagated in DMEM (30-2002, ATCC) with 10 % fetal bovine serum and pen-strep. Myoblast differentiation was induced by replacing the media with DMEM supplemented with 10 % horse serum at 120 hours post seeding, when cells reached 90 % confluence. At 120 hours post-seeding, cells were treated with siRNA Acly or control siRNA (Cultek) and were treated with 1 mM HC. Samples were snap frozen at different time points and maintained at -80 ºC until RNA was isolated.

**Semi-quantitative RT–PCR**

Total RNA was isolated from frozen tissues using PRImeZOL™ Reagent (AN1100, Canvax, Córdoba, Spain). RNA concentration and quality were determined using a NanoDrop® Spectrophotometer ND-100. Total RNA (0.5-2 µg) was used to synthesize cDNA with the iScript™ cDNA Synthesis Kit (Bio-Rad Laboratories). Primer sequences are presented in Table S1. The mRNA expression was calculated by the 2-ΔΔCT method and expression was normalized to the expression of Rps29.

**Oxygen consumption and extracellular acidification rates**

Mitochondrial bioenergetics on AML12 cells were measured using an XF24 Extracellular Flux Analyzer (Agilent). After 16 h of treatment with ACLY inhibitors, cells were washed with Seahorse assay media supplemented with 10 mM glucose, 1 mM pyruvate, and 2 mM glutamine, pH 7.2 (Agilent). Cells were incubated in a CO_2_-free incubator at 37 °C for 1 hour. Then, OCR and ECAR were determined in basal conditions and through consecutive injections of oligomycin (4 μM) at minute 27, carbonyl cyanide 4-(trifluoromethoxy) phenylhydrazone (FCCP; 2 μM) at minute 52, rotenone (1 μM) at minute 78, and antimycin A (5 μM) at minute 104.

**Acid histone extraction**

Liver samples (∼60 mg) were lysed in 0.3 ml of PBS containing 0.5 % Triton X 100 (v/v), 0.02 % (w/v) sodium azide, 10 μM trichostatin, 10 mM nicotinamide, 50 mM sodium butyrate and protease/phosphatase inhibitors P0044, P5725, P8340 (Sigma-Aldrich). Lysates were incubated for 10 minutes at 4 ºC with gentle rotation and the mixture was centrifuged at 2000 rpm for 10 minutes at 4 ºC. The supernatant was removed and the pellet was resuspended in 100 μl of 0.2 N HCl and incubated overnight at 4 ºC with gentle rotation. Then, samples were centrifuged at 2000 rpm for 10 minutes at 4 ºC and the supernatant harvested. Finally, HCl was neutralized with NaOH 0.2 N and samples were stored at -80 ºC until used.

**Statistics and reproducibility**

The statistical analysis was performed using SigmaPlot 14.5 (SigmaPlot, Barcelona, Spain) and GraphPad prism 7 (GraphPad Software Inc, San Diego, CA). Statistical tests are reported in figure legends. Normality was assumed in statistics. Dunn´s post hoc test was using in Kruskal Wallis non-parametric ANOVA. Bonferroni or Tukey post hoc test were used in ANOVA. Data are shown as means ± standard error of the mean (SEM). Significance is reported at p ≤ 0.05.

**REFERENCES**

Bonomini, F., Rodella, L. F., Moghadasian, M., Lonati, C., & Rezzani, R. (2013). Apolipoprotein E deficiency and a mouse model of accelerated liver aging. Biogerontology, 14(2), 209–220. https://doi.org/10.1007/s10522-013-9424-9

Hara, A., & Radin, N. S. (1978). Lipid extraction of tissues with a low‐toxicity solvent. Analytical Biochemistry, 90(1), 420–426. https://doi.org/10.1016/0003-2697(78)90046-5

Leger, M., Quiedeville, A., Bouet, V., Haelewyn, B., Boulouard, M., Schumann‐Bard, P., & Freret, T. (2013). Object recognition test in mice. Nature Protocols, 8(12), 2531–2537. https://doi.org/10.1038/nprot.2013.155

Liu, J., Xu, M. Y., Wu, J., Zhang, H., Yang, L., Lun, D. X., Hu, Y. C., & Liu, B. (2021). Picrosirius‐polarization method for collagen fiber detection in tendons: A mini‐review. Orthopaedic Surgery, 13(3), 701–707. https://doi.org/10.1111/os.12627

MacDonald, M. J., Longacre, M. J., Warner, T. F., & Thonpho, A. (2013). High level of ATP citrate lyase expression in human and rat pancreatic islets. Hormone and Metabolic Research, 45(5), 391–393. https://doi.org/10.1055/s-0032-1329987

Nash, A. M., & Frankel, E. N. (1986). Limited extraction of soybeans with hexane. Journal of the American Oil Chemists' Society, 63, 244–246. https://doi.org/10.1007/BF02546147

Vinel, C., Lukjanenko, L., Batut, A., Deleruyelle, S., Pradère, J. P., Le Gonidec, S., Dortignac, A., Geoffre, N., Pereira, O., Karaz, S., Lee, U., Camus, M., Chaoui, K., Mouisel, E., Bigot, A., Mouly, V., Vigneau, M., Pagano, A. F., Chopard, A., … Dray, C. (2018). The exerkine apelin reverses age‐associated sarcopenia. Nature Medicine, 24(9), 1360–1371. https://doi.org/10.1038/s41591-018-0131-6

Vue, Z., Garza‐Lopez, E., Neikirk, K., Katti, P., Vang, L., Beasley, H., Shao, J., Marshall, A. G., Crabtree, A., Murphy, A. C., Jenkins, B. C., Prasad, P., Evans, C., Taylor, B., Mungai, M., Killion, M., Stephens, D., Christensen, T. A., Lam, J., … Hinton, A., Jr. (2023). 3D reconstruction of murine mitochondria reveals changes in structure during aging linked to the MICOS complex. Aging Cell, 22(12), e14009. https://doi.org/10.1111/acel.14009
